# Synergistic lipid compositions for albumin receptor mediated delivery of mRNA to the liver

**DOI:** 10.1038/s41467-020-16248-y

**Published:** 2020-05-15

**Authors:** Lei Miao, Jiaqi Lin, Yuxuan Huang, Linxian Li, Derfogail Delcassian, Yifan Ge, Yunhua Shi, Daniel G. Anderson

**Affiliations:** 1 Koch Institute for Integrative Cancer Research, Massachusetts Institute of Technology, Cambridge, MA 02142 USA; 2 Ming Wai Lau Centre for Reparative Medicine, Karolinska Institutet, Hong Kong, China; 3 Department of Anesthesiology, Boston Children’s Hospital, 300 Longwood Ave, Boston, MA 02115 USA; 4 Division of Regenerative Medicine and Cellular Therapy, University of Nottingham, Nottingham, NG7 2RD UK; 5 Department of Molecular Biology, Massachusetts General Hospital, Cambridge, MA 02114 USA; 6 Department of Genetics, Harvard Medical School, Cambridge, MA 02115 USA; 7 Department of Chemical Engineering, Massachusetts Institute of Technology, Cambridge, MA 02142 USA; 8 Institute for Medical Engineering and Science, Massachusetts Institute of Technology, Cambridge, MA 02139 USA; 9 Harvard-MIT Division of Health Science and Technology, Massachusetts Institute of Technology, Cambridge, MA 02139 USA

**Keywords:** Biomedical materials, DNA and RNA, Drug delivery

## Abstract

Lipid-like nanoparticles (LNPs) have potential as non-viral delivery systems for mRNA therapies. However, repeated administrations of LNPs may lead to accumulation of delivery materials and associated toxicity. To address this challenge, we have developed biodegradable lipids which improve LNPs clearance and reduce toxicity. We modify the backbone structure of Dlin-MC3-DMA by introducing alkyne and ester groups into the lipid tails. We evaluate the performance of these lipids when co-formulated with other amine containing lipid-like materials. We demonstrate that these formulations synergistically facilitate robust mRNA delivery with improved tolerability after single and repeated administrations. We further identify albumin-associated macropinocytosis and endocytosis as an ApoE-independent LNP cellular uptake pathway in the liver. Separately, the inclusion of alkyne lipids significantly increases membrane fusion to enhance mRNA release, leading to synergistic improvement of mRNA delivery. We believe that the rational design of LNPs with multiple amine-lipids increases the material space for mRNA delivery.

## Abstract (web summary)

Lipid-like nanoparticles have applications as non-viral delivery systems for mRNA. Here, the authors develop biodegradable lipids with improved clearance and reduced toxicity.

## Introduction

Messenger RNA (mRNA) is being broadly investigated as a tool for vaccination^1^, protein replacement therapies^2^, and genome editing^3–5^. The clinical translation of mRNA therapies is limited in part by the need for improved delivery systems^5^; those which can address challenges associated with mRNA’s chemical instability and potential immunogenicity, and limited intracellular delivery^6^. Lipid nanoparticles (LNPs) have been identified as a leading system for siRNA delivery and are now being used with mRNA^7^. To provide a therapeutic effect, LNPs must both deliver functional mRNA to the cytoplasm of target cells, and be tolerated by the patients, to enable repeated administration at therapeutically relevant doses^8^.

LNPs are often formulated with phospholipids, cationic/ionizable amino lipids, poly(ethylene) glycol (PEG)-lipid, and cholesterol^9^. The cationic/ionizable amino lipids are critical for mRNA transfection, and can be synthesized using rational design approaches to systematically vary the lipid head and tail structures^8,10,11^. Alternatively, combinatorial methods have been used to generate libraries of lipid-like materials and identify non-traditional lipid structures^9^. To improve the endosomal release and cytoplasmic delivery of mRNA, fusogenic lipids such as dioleoylphosphatidylethanolamine (DOPE) have been incorporated into the LNPs^12,13^. DOPE tends to form the inverse hexagonal phase (H_II_), which destabilizes endosomal membrane and facilitates the release of mRNA^12^. In addition, decreasing the degree of saturation of the hydrophobic tails of the ionizable lipid has also been reported to contribute to forming of H_II_ phase. An increasing number of double bonds corresponds with an increasing propensity to form the non-bilayer phase^12,14,15^. Therefore, linoleic acid derived tails have been incorporated into lipids to achieve an enhanced siRNA/mRNA delivery efficiency^10^. Many of these materials are not biogradable. To improve the biocompatibility of lipid-like materials, ester linkages and disulfide bonds can be introduced into the lipid backbones^8,11,16–18^. However, biodegradable linkages can also introduce instability and reduce protein expression in hepatocytes^8,11,17,18^.

Here, we develop synergistic lipid formulations, which combines lipids that demonstrate good cellular uptake with biodegradable alkyne lipids which enhance endosomal escape and systemic tolerability. We have used these synergistic formulations to generate an LNP with enhanced intracellular protein expression. We first developed a library of biodegradable alkyne lipids with moderate intrinsic mRNA delivery efficacy, comparable to delivery using DLin-MC3-DMA (MC3) LNPs. We then combined these with unmodified lipids, and demonstrate that these lipids act synergistically to increase mRNA delivery ~10 times higher protein expression than MC3 alone and 2–5-fold higher than cKK-E12 alone. We further investigated the mechanism of this enhanced delivery, and identified an ApoE independent intracellular delivery pathway facilitated by albumin coating of LNPs. We also demonstrate that the incorporation of alkyne structures rather than double bonds in the hydrophobic tails of ionizable lipids could increase the fusogenicity, facilitating endosomal escape. The combinatorial formulation significantly reduces the amount of unmodified lipids used in the formulation, and therefore also increases the systemic tolerability of the formulation.

## Results

### Synthesis of biodegradable alkyne lipids

**Fig. 1:**
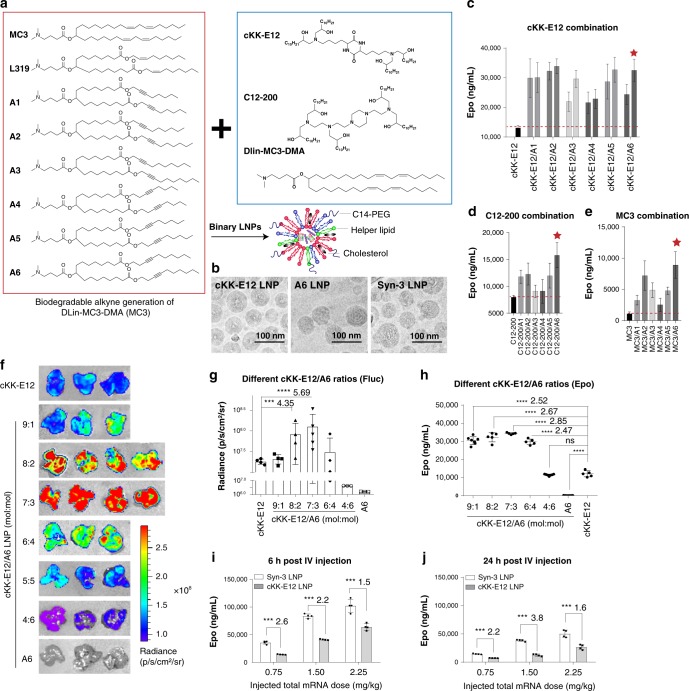
LNPs containing multiple ionizable lipids demonstrate synergistic mRNA expression in vivo. **a** Design of biodegradable alkyne lipids and layout of the combination strategy. **b** Cryo-EM image and schematic illustration of mRNA loaded LNPs. **c**–**e** I.v. injection of hEPO mRNA loaded LNPs containing two different ionizable lipids (0.75 mg/kg of hEPO mRNA). **c** cKK-E12 and alkyne lipid combinations were evaluated at two molar ratios (left: 5:5, right: 7:3); combination of C12-200 and MC3 with alkyne lipids were evaluated at molar ratio 7:3, respectively. Formulation includes a composition of ionizable lipid:DOPE:cholesterol:C14-PEG2000 at the molar ratio of 35:16.0:46.5:2.5. Six hours after injection, hEPO protein in the serum was detected and quantified using hEPO ELISA. Red stars highlight the top-performing synergistic LNPs (*n* = 4/group). **f**, **g** IVIS images and quantification of luciferase expression. mRNA encoding firefly luciferase mRNA (Fluc mRNA) was formulated with LNPs composed of various molar ratios of cKK-E12 and A6. A molar ratio of cKK-E12:A6 at 7:3 (Syn-3) demonstrated the most robust enhancement in Fluc protein expression in the liver (*n* = 4 in cKK-E12, 9:1, 8:2, 6:4, *n* = 5 in 7:3 group, *n* = 3 in 4:6 and A6 groups, representative images were shown in **f**). **h** The optimal ratio of cKK-E12:A6 in the LNP formulation was also evaluated using hEPO mRNA. The optimal ratio was also found to be 7:3. **i**, **j** Dose-dependent and time-dependent protein expression using Syn-3 (cKK-E12:A6 is 7:3) LNPs was compared with cKK-E12 LNPs, and demonstrated superior gene expression in vivo. Data are presented as mean ± SD. ***P < 0.001, ****P < 0.0001, Student’s T-test comparing to cKK-E12 group.

The *cis*-double bond derivative of linoleic acid is a key component in the lipid tail of MC3^10^. Within the body, this moiety can undergo desaturation into linolenic acid derivatives or acetylation into crepenynic acid derivatives (Supplementary Fig. 1)^19^. Based on the structure of the natural acetylated alkyne metabolites (Crepenyic acid) of linoleic acids and the original chemical structure of MC3, we designed six alkyne lipids (Fig. 1a) featuring the same dimethylamino head group with different tails. We further introduced ester linkages into the hydrocarbon tails to allow enzymatic hydrolysis of lipids, which liberates a more hydrophilic carboxylic acid and an alcohol in tissues for accelerated clearance^8,11^. The structures of these newly synthesized lipids are similar to the biodegradable lipid L319^11^. Our modifications substituted the L319 alkene for an alkyne, and moved the ester linkages to a new position.

Our results suggest that substituting the alkene (L319) with an alkyne significantly improved both in vivo and in vitro mRNA delivery efficiency in 4 of the 6 modified lipid nanoparticles with different cholesterol % (Supplementary Figs. 2 and 3). Interestingly, in vitro some of these alkyne lipids were able to outperform MC3, cKK-E12 and C12-200, which have been evaluated for clinical use (Supplementary Fig. 3). However, these trends were not conserved in in vivo tests (Supplementary Figs. 2 and 3). Given the enhanced transfection observed in vitro and somewhat impaired in vivo delivery, we next explored their use as helper lipids in LNPs formulated with MC3, cKK-E12, and C12-200.

### LNPs formulated with biodegradable and non-biodegradable amine lipids

To examine synergistic effects, we encapsulated mRNA encoding human erythropoietin (hEPO) in LNPs formulated from alkyne lipids mixed with MC3, cKK-E12, or C12-200 lipids, respectively, using DOPE as a helper lipid (Fig. 1a). The addition of biodegradable alkyne lipids enhanced hEPO production as compared with the original mono-lipid LNP formulation (Fig. 1b–d). In particular, newly designed alkyne lipid A6 demonstrated a significant improvement in transfection efficiency (~8.5, ~2.0, and ~2.5-fold higher than the original MC3, C12-200 and cKK-E12 containing LNPs, respectively) (Fig. 1b–d). The cKK-E12 and A6 combination demonstrated the most robust hEPO production in vivo, and so we used these two lipids as a model system to explore the optimal ratio for synergistic mRNA delivery.

We kept the overall ionizable lipid concentration at a constant ratio within the LNPs, and varied the ratio of cKK-E12 to A6 in this component. We observed a ratio-dependent synergy of the cKK-E12/A6 LNPs delivering either mRNA encoding Firefly luciferase (Fluc) or hEPO. When the lipid component contained cKK-E12 and A6 at a relative molar ratio of 7:3 (Syn-3 LNPs, Table 1), hEPO and Fluc production was highest, approximately 2.52 and 5.69-fold higher than the original, mono-lipid formulations (Fig. 1e–g, Table 1). At a fixed ratio of 7:3, the synergistic LNPs exhibited superior gene expression in a dose- and time-dependent manner (Fig. 1h, i).

**Table 1:** Labels for LNPs containing two amine lipids.

Synergistic LNP labels	cKK-E12/A6 (mol:mol)
Synergistic-1 (Syn-1)	9:1
Synergistic-2 (Syn-2)	8:2
Synergistic-3 (Syn-3)^a^	7:3
Synergistic-4 (Syn-4)	6:4
Synergistic-5 (Syn-5)	4:6

^a^The optimal formulation with strongest synergy.

We observed maximal protein expression in the liver, followed by the spleen. To identify protein expressing cells within the liver, we used loxP-flanked tdTomato reporter mice^20^, which express tdTomato upon delivery of Cre-recombinase mRNA (Cre mRNA). Mice were injected with Cre mRNA encapsulated in cKK-E12 LNPs or Syn-3 LNPs. We found that both hepatocytes and major non-parenchymal cells (i.e., macrophages, endothelial cells) expressed tdTomato, with parenchymal hepatocytes representing ~80% of tdTomato positive cells. The percentage of hepatocytes expressing tdTomato increased from 53.4 to 73.4% when A6 was included in the LNP formulation (Supplementary Fig. 4). As hepatocytes are a major target for mRNA expression, we used primary hepatocytes to evaluate the behavior of LNPs in vitro. Interestingly, we found that the addition of serum protein to the culture medium (to mimic physiological conditions) significantly improved the delivery of mRNA using cKK-E12 containing LNPs to primary hepatocytes at two different cholesterol % formulations (Supplementary Fig. 5), indicating that serum factors or proteins play a role in the delivery of mRNA to the liver.

### Albumin is the major protein in the synergistic LNPs corona

**Fig. 2:**
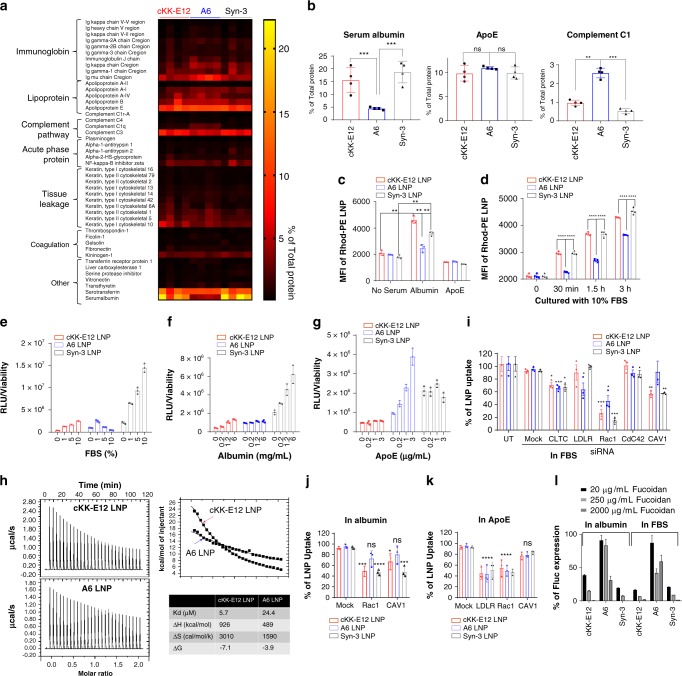
Protein corona facilitates the binding and internalization of cKK-E12 containing LNPs to primary hepatocytes. a Proteomics analysis of protein coronas isolated from three types of LNPs (*n* = 4/group). Quantification of % of major proteins are shown in (**b**). **c**, **d** Uptake of Rhod-PE labeled LNPs in primary hepatocytes in the presence or absence of selected proteins (n = 4/group). One-way ANOVA. **e**, **g** Expression of luciferase mRNA 6 h post transfection of primary hepatocytes in different protein pretreatment conditions (*n* = 3/group). **h** ITC data estimates the binding between LNPs and albumin. One-wat ANOVA. **i**–**k** siRNA knockdown of certain endocytosis proteins demonstrates the mechanisms of endocytosis of different LNPs in the presence or absence of serum proteins (*n* = 3/group). Student’s T-test comparing to Mock group. **l** The function of albumin in mediating the endocytosis was confirmed using the albumin receptor inhibitor fucoidan (*n* = 4/group). Data are presented as mean ± S.D. *P < 0.05; **P < 0.01; ***P < 0.001; ****P < 0.0001. Statistical methods used for *P* value calculates are listed above in the legend.

Serum proteins are the major biological component of the corona coating formed on NPs following exposure to biological fluids^21^. Formation of a protein corona on our LNPs was confirmed using particle size measurements; the average diameter of LNPs increased ~20 nm after incubation with mouse serum for 1 h (Table 2. Supplementary Fig. 6). No aggregates were found when incubating LNPs with 10% serum saline 4 h after incubation. However, decomposition of A6 LNPs were observed at 4 h, which may be attributed to the degradation of biodegradable A6 lipids in serum (Supplementary Fig. 6). We then used a size-exclusion column to isolate the “hard protein corona” with higher binding affinity of cKK-E12 LNPs, A6 LNPs, and Syn-3 LNPs (Fig. S7)^22,23^ and used proteomics to analyze the composition of proteins on LNPs (Supplementary Table 1). Forty-six proteins were detected and quantified (Fig. 2a) on the LNPs. These proteins are involved in the function of complement activation, immune responses, coagulation, acute phase response and lipid metabolism (Fig. 2a, Supplementary Table 1). Previous studies identified apolipoproteinE (ApoE) as an important protein for LNPs; which can mediate LNP delivery to the liver^24,25^. Consistent with this, we identified ApoE in the corona of all three LNPs, and as the dominant plasma protein in the corona of A6 LNPs. Although the amount of ApoE appears similar in the corona of all three LNPs, the corona of cKK-E12 and Syn-3 LNPs is dominated by a 5–6-fold increase in serum albumin concentration, with ApoE composing a much smaller fraction of the corona of these LNPs (Fig. 2b).

**Table 2:** Characterization of LNP formulations.

	Fluc mRNA					hEPO mRNA			
LNPs	pKa	D (nm)	PDI	EE (%)	Zeta (mV)	D (nm)	PDI	EE (%)	In FBS (1 h), D (nm)
cKK-E12	6.51 ± 0.12	86.7 ± 0.5	0.17	65.5 ± 3.8	−5.5 ± 1.2	82.5 ± 1.8	0.13	56.5 ± 3.3	101.7 ± 3.3
A6	6.65 ± 0.15	85 ± 1.2	0.10	75.2 ± 3.1	−1.1 ± 0.8	78.2 ± 2.1	0.16	81.5 ± 2.5	101.3 ± 10.3
Syn-3	6.78 ± 0.14^a^	99.5 ± 0.3	0.07	72.5 ± 2.2	−2.1 ± 1.3	91.2 ± 0.5	0.13	71.2 ± 3.2	115.2 ± 4.5

^a^Apparent pKa.

### Serum albumin and ApoE-mediated cellular uptake

To evaluate whether the ratio of albumin and ApoE would affect cellular uptake and intracellular trafficking of these LNPs, LNPs were pre-labeled with fluorescent dye (either with rhod-PE on the lipid particles or with Cy5-mRNA) and cultured in a protein-supplemented media. In serum-free media, cellular uptake remained the same for all three LNPs (Fig. 2c, Supplementary Fig. 8); whereas in serum-containing media, the hepatocytes uptake of cKK-E12 and Syn-3 LNPs was significantly increased as compared with A6 LNPs (Fig. 2d). By treating hepatocytes with LNPs incubated in an albumin-rich or ApoE solution (Fig. 2c, Supplementary Fig. 8), we demonstrated that albumin plays a key role in the cellular uptake of cKK-E12 containing LNPs. Previous work has described ApoE-mediated cellular uptake of siRNA LNPs, however, in our case the role of ApoE is less significant. This could be due to either saturation of ApoE secretion from hepatocytes masking LNP-mediated ApoE uptake effects, or the intrinsic difference between mRNA and siRNA LNP formulations^24,25^. Therefore, we hypothesize that LNPs coated with serum albumin may facilitate the delivery of mRNA to the liver via an ApoE “independent” pathway.

To further evaluate this hypothesis, we studied the protein expression of cKK-E12, A6, and Syn-3 LNPs containing Fluc mRNA in primary hepatocytes in the presence or absence of albumin, ApoE, or serum. Consistent with the uptake study and studies using ionizable LNPs^11,25^, protein expression with the A6 mRNA LNP was found to be independent of albumin or serum, but significantly enhanced by ApoE (Fig. 2e–g). On the contrary, for cKK-E12 LNPs or Syn-3 LNPs, increasing the concentration of ApoE in the media slightly enhanced gene expression (Fig. 2e–g) whereas increasing the concentration of serum and albumin dramatically increased gene expression 4–5-fold (Fig. 2e, f).

To further investigate the interaction between albumin and LNPs, the thermodynamics of albumin adsorption on LNP surfaces was determined using isothermal titration calorimetry (ITC). Although, a relatively weak interaction was observed for both cKK-E12 LNPs and A6 LNPs with human albumin (HSA)^26,27^, HSA adsorbed on cKK-E12 LNPs (Kd: 5.7 μM) demonstrated a 5-fold higher association constant as compared with A6 LNPs (Kd: 24.4 μM). The enthalpy of HSA binding to CKK-E12 LNPs (926 kcal/mol) was 2-fold higher than A6 LNPs (489 kcal/mol) (Fig. 2h).

Previous studies have shown that albumin-like moieties, including albumin-NPs, can bind preferentially to the glycoprotein scavenger receptors gp30 and gp18. This interaction facilitates endocytosis via the caveolae mediated pathway or via macropinocytosis^28^. To test whether our LNPs were internalized via an albumin-mediated pathway following albumin coating, we evaluated several cellular internalization pathways. We depleted key endocytic regulators using siRNA in primary hepatocytes (Fig. 2i), and delivered our LNPs. A6 LNPs were mostly internalized through Ras-related C3 botulinum toxin substrate 1 (RAC-1)-mediated macropinocytosis and partially through ApoE-low-density lipoprotein receptor (LDLR)-mediated endocytosis (Fig. 2i). In contrast, cKK-E12 LNPs and Syn-3 LNPs relied on caveolae (CAV)-mediated endocytosis (through CAV1) (Fig. 2i). This was observed when incubating cKKE12 containing LNPs with albumin (Fig. 2j), but not with ApoE (Fig. 2k), confirming that albumin-associated macropinocytosis and endocytosis facilitates the uptake of LNPs containing cKK-E12. Finally, the mRNA delivery efficiency of cKK-E12 and Syn-3 LNPs was reduced in a dose-dependent manner during co-culture with a strong gp18 and gp30 competitor, fucoidan^29,30^, further validating our hypothesis (Fig. 2l). Overall, the above study demonstrates that the addition of A6 lipids in LNP formulations does not significantly affect the mechanism of endocytosis. In contrast, cKK-E12 lipids govern the mechanism of cellular uptake; through endocytosis of the Syn-3 LNPs via an albumin-loaded protein corona. Syn-3 lipids with optimal ratios of cKK-E12 can maintain their stability and facilitate albumin binding, and are internalized through these endocytic pathways.

### Formulations with optimal membrane fusion and endosomal escape

**Fig. 3:**
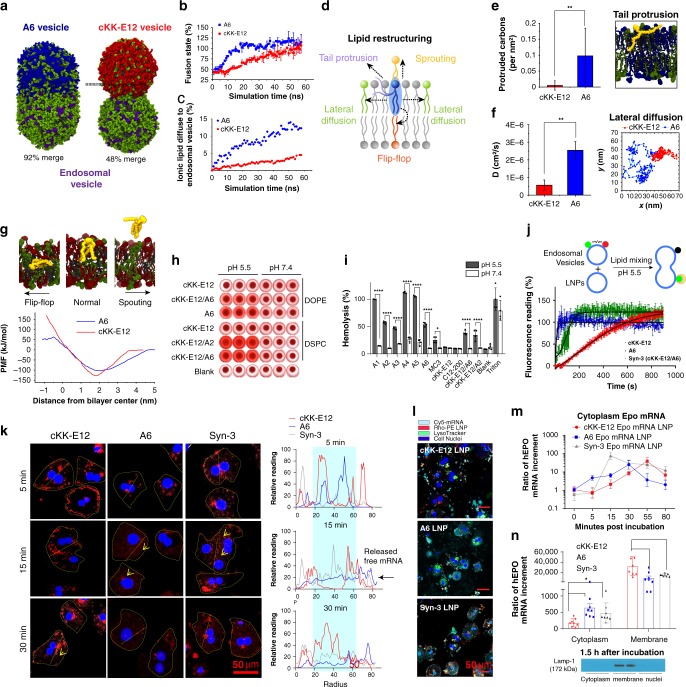
Alkyne lipids facilitate the endosomal fusion and release of mRNA from LNPs. **a** Schematic of fusion between A6 (blue) or cKK-E12 (red) vesicles and endosomal vesicles by MD simulation. DOPE and DOPC are in green, LBPA is in purple, cholesterol is in gray. **b** Estimated vesicle merging states between A6 or cKK-E12 and endosomal vesicles. Merging state is defined as the ratio between the neck area and the largest intersection area of the endosomal vesicle (Error bars: variation of the neck area). **c** Percentile of A6 or cKK-E12 lipid mixed in endosomal vesicles during fusion simulation. **d** Schematic of lipid restructuring movements in LNPs during fusion. **e** MD calculations of lipid tail protrusion in cKK-E12 and A6 membranes (*n* = 1000). Student’s T-test. Panel on the right: a snapshot of A6 lipid protruding out the membrane with one tail. **f** Lateral diffusion coefficient of A6 and cKK-E12 lipid in singular membranes calculated from the mean square displacement using linear regression (*n* = 5000) (Error bars: errors between individual lipids). Student’s T-test. Penal on the right: trajectory of A6 or cKK-E12 lipid within the singular membrane over 40 ns simulation. **g** Free energy profile of A6 and cKK-E12 lipid sprouting and flip-flop action. **h**, **i** hemolysis analysis of variable LNPs in acidic and neutral pH conditions (*n* = 4), Student’s T-test. **j** Fluorescence resonance energy transfer (FRET) based membrane fusion assay. Rhodamine-PE and NBD-PE dual labeled endosomal vesicles (donor vesicles) were mixed with non-fluorescent labeled cKK-E12, A6, or Syn-3 LNPs. NBD fluorescence was monitored at 540 nm with excitation at 465 nm upon mixing. Mixing of un-labeled vesicles were subtracted as blank. **k** Time-lapse images of released free mRNA in primary hepatocytes treated with Cy5-mRNA LNPs in 10% serum. Hepatocytes were highlighted in yellow dotted circles. Yellow arrows indicate released free fluorescent mRNA observed within cytoplasm. Quantifications presented on right were based on the fluorescence intensity around the straight dotted yellow lines across hepatocytes. Blue rectangle highlights the fluorescence distributed near cell nuclei. **l** Hepatocytes were incubated with Cy5-mRNA-encapsulated Rhob-PE-labeled LNPs for 1 h in 10% serum. Lyso-endosome system was stained with LysoTracker green. The cytoplasmic distribution of LNPs and mRNA were visualized using confocal microscope. **m**, **n** Subcellular fractionation of hEPO mRNA from cytoplasm or membrane containing vehicles. **m** Time-lapse release of free hEPO mRNA into cytoplasm (*n* = 8). **n** At 1.5 h after incubation, the distribution of hEPO mRNA in the cytoplasmic compartment and membrane associated compartment was quantified by rt-PCR (*n* = 4), Student’s T-test. The subcellular isolation was confirmed by membrane associated marker Lamp-1 staining. All data are presented as mean ± SD. *P < 0.05, **P < 0.01, ***P < 0.001, ****P < 0.0001.

The cKK-E12 and Syn-3 LNPs showed similar levels of intracellular uptake (Fig. 2d), yet protein expression was superior in the Syn-3 LNP system containing the lipid A6 (Fig. 2e). This suggests that inclusion of A6 lipids in the LNP may increase expression by some other mechanisms. Endosomal cargo release is another “bottle neck” in transgene expression, and so we tested the effect of the A6 lipid on intracellular release of mRNA-encapsulated LNPs from endosomes/lysosomes. LNPs can destabilize and escape endosomes through fusion with the endosomal membrane^7,31^. Fusion results in disintegration of both entities and the release of the encapsulated genes^3,7,32^. We hypothesized that addition of A6 to the cKK-E12 LNPs may improve membrane fusion and destabilization relative to LNPs formulated from cKKE12 alone. To test our hypothesis, we performed atomistic level vesicle-vesicle fusion simulations (OPLS-AA force field with explicit TIP4P water model^33^), using computationally constructed A6 or cKK-E12 vesicles and endosomal vesicles (42% DOPE, 17% 1,2-dioleoyl-sn-glycero-3-phosphocholine (DOPC), 13% lysobisphosphatidic acid (LBPA), and 28% cholesterol)^34^. The topology of LBPA and construction of the bilayer and vesicle system can be found in Supplementary Table 2 and Supplementary Figs. 9–20. According to our model, we found that the fusion between A6 and endosomal vesicles was ~3 times faster than that between cKK-E12 and endosomal vesicles (Fig. 3a–c, and Supplementary Figs. 21–23). Of note, the mixing of A6 into endosomal vesicles is more rapid as compared with cKK-E12. In contrast, LBPA from endosomal vesicle has the same level of mixing into both A6 and cKK-E12 vesicles (Supplementary Fig. 24).

To evaluate the fusion and internalization of these LNPs, we compared the ability of A6 and cKK-E12 to integrate within bilayer membranes using three parameters: tail protrusion, lateral diffusion, lipid sprouting, and flip-flopping (vertical movement) (Fig. 3d). Prior studies have suggested that establishing fusion stalks that connect the opposing membranes is crucial to initiate fusion, and that hydrophobic tail protrusion in the polar membrane surface is a key step required to form fusion stalks^35,36^. We defined a simulation protrusion event as any carbon in the lipid tail protruding more than 0.1 nm beyond the average height of DOPE phosphorus atoms in the leaflet^35^. We found the frequency of A6 tail protrusion was ~10-fold higher than cKK-E12 in membranes that only feature one type of lipids (Fig. 3e), suggesting an increased likelihood of forming an early endosomal membrane stalk structure with A6 LNPs. We further noticed that inclusion of a small amount of A6 into a cKK-E12 rich membrane (Syn-3) could increase the protrusion of both lipids (Supplementary Fig. 25). The simulation also indicated that the lateral diffusion coefficient of A6 was much higher than that of cKK-E12 (Fig. 3f), explaining the observed fast lipid mixing of A6 into endosomal vesicles compared with cKK-E12 (Fig. 3c). We calculated the free energy of lipid sprouting and flip-flopping to estimate the ability of lipids to move vertically inside the membrane. These calculations indicate that it takes ~130 kJ/mol for cKK-E12 to partition into bulk water (sprout) whereas the energy required for A6 sprouting is <90 kJ/mol (Fig. 3g). In addition, cKK-E12 shows a steeper energy barrier than A6, indicating that cKK-E12 is less vertically mobile and is more stable in its equilibrium position. Interestingly, energy required for the “helper” lipid DOPE to sprout is also higher in cKK-E12 bilayer than in A6 bilayer (Fig. S26). For lipid flip-flop, on the other hand, cKK-E12 shows a much higher energy barrier than that of A6, indicating that cKK-E12 can rarely flip between leaflets. Thus, the tail protrusion, diffusion coefficient, and free energy of lipid sprouting and flip-flop action all suggest that A6 affords preferential fusion initiation and is likely to have faster fusion kinetics compared with cKKE12.

The relative capacity for LNP-mediated membrane fusion and destabilization was further investigated using a red blood cell (RBC) hemolysis assay. Due to the similarities in the lipid bilayer structure between RBCs and endosomes, RBCs are a model system to test membrane fusion^37^. Consistent with our model and hypothesis, alkyne lipids induced robust RBC hemolysis at acidic endosomal pH. In contrast, there was almost no hemolysis observed using cKK-E12 and C12-200 LNPs at the concentration tested (Fig. 3h, i). As expected, inclusion of A6 lipids into cKK-E12 formulations improved the fusion of LNPs at acidic pH (Fig. 3h, i). Endosomal membrane fusion was further measured by a well-established fluorescence resonance energy transfer (FRET) based membrane fusion assay^38^. Using endosomal vesicles as membrane donors, we observed efficient membrane fusion when combined with A6 membranes at acidic pHs. Inclusion of A6 alkyne lipids into the synergistic Syn-3 formulation (including cKK-E12 and A6) increased the overall fusion of the particles (Fig. 3j). To correlate the fusion kinetics with endosomal escape, we used confocal microscopy to follow the kinetics of Cyanine-5 (Cy5)-labeled mRNA escape in primary hepatocytes (Fig. 3k, Supplementary Fig. 27). Particles were incubated with cells on ice for 30 min to allow sufficient binding. Internalization was initiated when particles were removed and cells were transferred to 37 °C. Despite a lower amount of LNP binding on the cell surface, A6 LNPs demonstrated a rapid cytoplasmic release of Cy5-labeled mRNA approximately within 15 min after internalization. In contrast, cKK-E12 LNPs were preferentially bound to hepatocytes, however upon internalization, most of the mRNA aggregated in vesicles. We observed a gradual increase in fluorescence within the cytoplasm 30 min after internalization. Inclusion of A6 lipidoids into our Syn-3 formulations accelerated mRNA release, which was observed within 15 min of incubation. We further co-stained the LNP treated hepatocytes (1 h incubation) with Lysotracker green (Fig. 3l), confirming that the majority of mRNA LNP aggregates within hepatocytes were trapped in the endo-lysosome systems. We also used cellular fractionation to distinguish the mRNA released in the cytosol from that sequestered in the endolysosomal compartment (Fig. 3m, n). The amount of mRNA in the cytosol and endosomes was quantified with quantitative PCR following fractionation (Fig. 3m). Consistent with the microscopy results, cytosolic Epo mRNA was detected in cells treated with A6 5–15 min after incubation (Fig. 3m). Peak concentrations were reached at 25 min; and then decreased gradually over the next 50 min, which may be due to the gradual degradation of mRNA. Inclusion of A6 lipid into cKK-E12 formulations accelerated cargo release, with maximal release moving from 55 min (cKK-E12 LNPs) to 15 min (Syn-3 LNPs) after incubation. These results suggest that addition of A6 lipid into LNPs enhances intracellular endosomal release of LNPs. Despite this enhanced endosomal release, we noted that a very large fraction of mRNA is still trapped in the membrane systems within hepatocytes for all three treatment groups (Fig. 3l, m, Supplementary Fig. 28). These results are consistent with the fluorescent images as well as other literature reports^39^.

### Synergistic formulation for repeated mRNA delivery in vivo

**Fig. 4:**
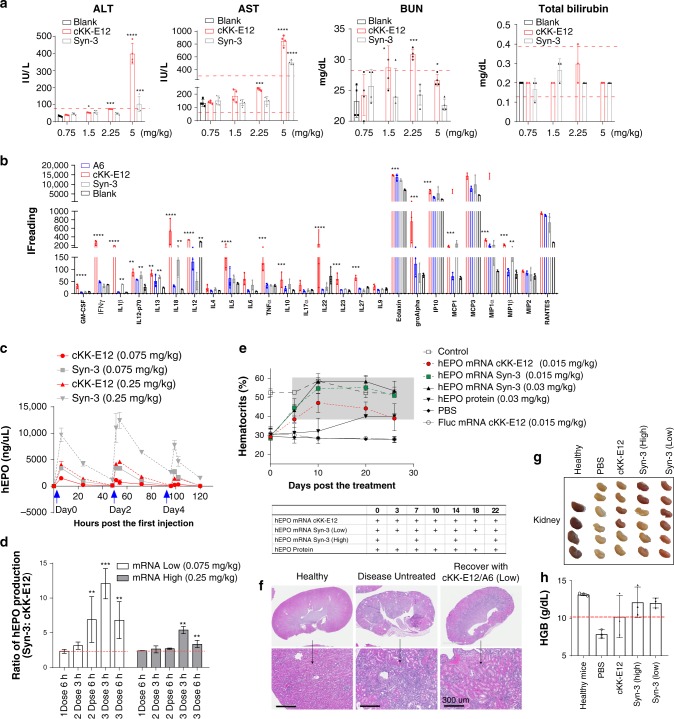
Tolerability and multiple dosing efficacy of synergistic LNPs. **a** Dose-dependent blood chemistry assay demonstrated decreased toxicity of Syn-3 LNPs as compared with cKK-E12 LNPs with relatively low values of AST, ALT, BUN, and total bilirubin (*n* = 4/group), Student’s T-test comparing to blank. **b** The multi-plex cytokine assay of blank LNPs (equivalent to 1.5 mg/kg mRNA LNPs) after single dose (*n* = 4/group), Student’s T-test comparing to blank. **c** mRNA expression kinetics after multiple dosing of mRNA containing LNPs. Protein expression ratio of Syn-3 LNPs were compared with mono cKK-E12 LNPs at different time points (*n* = 4). **d** Student’s T-test comparing to 1 Dose 6 h. **e** treatment of renal anemia by different forms of hEPO containing LNPs (*n* = 6–8). **f**, **g** Morphology and structure of kidney after treatments. **h** HGB levels were also compared in different groups (*n* = 6). Data are presented as mean ± SD. *P < 0.05, **P < 0.01, ***P < 0.001, ****P < 0.0001.

Next, we evaluated whether inclusion of biodegradable lipids improved LNP safety and tolerability. In a single-dose escalation study in healthy mice, we dosed cKK-E12 LNPs and Syn-3 LNPs at various mRNA concentrations. Twenty-four hours after intravenous injection, there were no significant changes in key liver (e.g., AST, ALT) and kidney (e.g., BUN, total bilirubin) toxicity markers using Syn-3 LNPs at low administration dosage (≤2.25 mg/kg) (Fig. 4a). This is in contrast to the toxicity profile of cKK-E12 LNPs containing mRNA, which showed a dose-dependent increase in AST, ALT, BUN, and total bilirubin (Fig. 4a). At a 2.25 mg/kg dose or higher of mRNA in cKK-E12 particles, AST and BUN were already close to or exceeded their normal range. Although the structure of particles without mRNA can be substantially different, we evaluated the immunogenicity of LNPs formulated without mRNA. After a single injection of blank cKK-E12 LNPs, we observed elevation of interleukins and chemokines (Fig. 4b). Notably, proinflammatory associated cytokines and chemokines, i.e., IFN, IL6, TNFα, groAlpha, and MCP1, were upregulated. This trend was not observed in A6 LNPs, and the level of inflammatory cytokines was reduced in the Syn-3 LNP system compared with cKK-E12 alone.

Next, we explored whether these synergistic LNP formulations (using the biodegradable lipid A6/cKK-E12 formulation) would lower toxicity associated with repeated dosing. After the third dose, Syn-3 LNPs induced lower immune stimulation compared with cKK-E12 LNPs (Supplementary Fig. 29A). Using Syn-3 LNPs also reduced AST levels compared with cKK-E12 LNPs illustrating the potential of applying Syn-3 LNPs for repeated dosing (Supplementary Fig. S29B). We then tested whether Syn-3 LNPs with improved tolerability could enhance protein expression after repeated injections. As can be seen from Fig. 4c, d, we observed consistent protein expression using two doses of Syn-3 LNPs containing hEPO mRNA, and a slight decrease of expression in the third dose. In contrast, we found that after the first injection of cKK-E12 LNPs, the protein expression decreased gradually following repeat doses.

To test the potential of Syn-3 mRNA LNPs as a protein replacement therapy, we used Epo mRNA LNPs to treat renal anemia. A renal anemia mouse model was developed using adenine treatment of C57BL/6 mice. Anemic mice were then treated with different hEPO mRNA-encapsulated LNPs (Fig. 4e). Notably, both 3-day interval injections and high-dose weekly injections of Syn-3 LNPs could maintain hematocrit and hemoglobin level during a 1-month treatment regime (Fig. 4e, h). Histological analysis showed that renal anemia was almost eliminated using this treatment regime (Fig. 4f–h). In contrast, cKK-E12 LNP treatment increased hematocrit level following the first two injections but this then gradually decreased. Overall, this data suggests that synergistic LNPs containing therapeutic mRNA could efficiently recover hematocrit levels and treat renal anemia.

## Discussion

Here, we have developed a partially biodegradable synergistic LNP with potent mRNA delivery efficacy and improved tolerability for both singular and repeated injection. Endocytosis and endosomal escape are two key processes controlling mRNA intracellular delivery efficiency^40^. Most LNPs were taken up by cells through macropinocytosis and receptor-mediated endocytosis into endosomal cargos, from where the mRNA can be released into cytoplasm^39^. Mechanistic studies suggest that synergistic LNPs containing two distinct lipid substructures can enhance mRNA delivery. cKK-E12-based lipids enhance serum stability and protein binding of the particles. We identified serum albumin as a major ApoE-independent pathway for cellular uptake of cKK-E12-based LNPs in hepatocytes. Further studies will be required to fully elucidate the effect of albumin or ApoE on endosomal release; however, we note that the efficacy of cKKE12 LNPs was limited by insufficient endosomal release. Separately, we demonstrated inclusion of an unsaturated alkyne group in the hydrophobic tail of the ionizable lipids could improve fusion with the endosomal membrane, and facilitate endosomal escape and cargo release. Using A6 lipids together with cKK-E12 in a single, synergistic LNP further enhances mRNA therapeutic efficacy. Based on this rationale, more efficient synergistic LNPs can be designed to boost the efficacy of well-established lipid libraries and enhance the delivery of mRNA therapeutics.

## Methods

### Lipid synthesis

Example of A6 synthesis route was shown in Supplementary Fig. 30, where the diacid 4 was synthesized as reported by Maier et al.^11^ previously. The last esterification step was also followed by the method reported by Maier et al.^11^. The distinct steps for all alkyne lipids (A1–A6) were described as below:

Synthesis of di(oct-2-yn-1-yl) 9-((4-(dimethylamino)butanoyl)oxy)heptadecanedioate (A1): The diacid 4 (0.43 g, 1 mmol) was dissolved in 20 mL of dichloromethane and 2-Octyn-1-ol (0.31 g, 2.44 mmol) was added to it followed by Hunig’s base (0.68 g, 4.9 mmol) and DMAP (12 mg). To this mixture EDCI (0.47 g, 2.44 mmol) was added and the reaction mixture was stirred at room temperature overnight. The reaction mixture was then diluted with CH_2_Cl_2_ (40 mL) and washed with saturated NaHCO_3_ (50 mL), water (60 mL), and brine (60 mL). The combined organic layers were dried over anhydrous Na_2_SO_4_ and solvents were removed in vacuo. The crude product thus obtained was purified by Combiflash Rf purification system (40 g silicagel, 0–10% MeOH in CH_2_Cl_2_) to afford the compound A1 as a colorless oil. Yield: 192.8 mg, 29.9%. ^**1**^H NMR (400 MHz, CDCl_**3**_) δ 4.87 (m, 1H), 4.15 (t, *J* = 7.0 Hz, 4H), 2.49 (tt, *J* = 7.0, 2.4 Hz, 4H), 2.32 (m, 8H), 2.24 (s, 6H), 2.16 (tt, *J* = 6.9, 2.3 Hz, 4H), 1.81 (m, 2H), 1.63 (m, 4H), 1.45 (m, 12H), 1.30 (s, 16H), 0.92 (t, *J* = 7.2 Hz, 6H). MS (Water Acquity LC-MS) (*m/z*): MW calc’d for C_39_H_68_NO_6_ (M + H^+^): 646.50, found: 646.42.

Synthesis of di(non-2-yn-1-yl) 9-((4-(dimethylamino)butanoyl)oxy)heptadecanedioate (A2): The diacid 4 (0.43 g, 1 mmol) was dissolved in 20 mL of dichloromethane and 2-Non-yn-1-ol (0.34 g, 2.44 mmol) was added to it followed by Hunig’s base (0.68 g, 4.9 mmol) and DMAP (12 mg). To this mixture EDCI (0.47 g, 2.44 mmol) was added and the reaction mixture was stirred at room temperature overnight. The reaction mixture was then diluted with CH_2_Cl_2_ (40 mL) and washed with saturated NaHCO_3_ (50 mL), water (60 mL), and brine (60 mL). The combined organic layers were dried over anhydrous Na_2_SO_4_ and solvents were removed in vacuo. The crude product thus obtained was purified by Combiflash Rf purification system (40 g silicagel, 0–10% MeOH in CH_2_Cl_2_) to afford the compound A2 as a colorless oil. Yield: 379.4 mg, 56.3%. ^1^H NMR (400 MHz, CDCl_3_) δ 4.87 (m, 1H), 4.15 (t, *J* = 7.0 Hz, 4H), 2.49 (tt, *J* = 7.0, 2.4 Hz, 4H), 2.32 (m, 8H), 2.24 (s, 6H), 2.16 (tt, *J* = 6.9, 2.3 Hz, 4H), 1.81 (m, 2H), 1.63 (m, 4H), 1.45 (m, 12H), 1.30 (s, 16H), 0.92 (t, *J* = 7.2 Hz, 6H). MS (Water Acquity LC-MS) (*m/z*): MW calc’d for C_41_H_72_NO_6_ (M + H^+^): 674.53, found: 674.60.

Synthesis of di(dec-2-yn-1-yl) 9-((4-(dimethylamino)butanoyl)oxy)heptadecanedioate (A3): The diacid 4 (0.43 g, 1 mmol) was dissolved in 20 mL of dichloromethane and 2-dec-yn-1-ol (0.38 g, 2.44 mmol) was added to it followed by Hunig’s base (0.68 g, 4.9 mmol) and DMAP (12 mg). To this mixture EDCI (0.47 g, 2.44 mmol) was added and the reaction mixture was stirred at room temperature overnight. The reaction mixture was then diluted with CH_2_Cl_2_ (40 mL) and washed with saturated NaHCO_3_ (50 mL), water (60 mL), and brine (60 mL). The combined organic layers were dried over anhydrous Na_2_SO_4_ and solvents were removed in vacuo. The crude product thus obtained was purified by Combiflash Rf purification system (40 g silicagel, 0–10% MeOH in CH_2_Cl_2_) to afford the compound A3 as a colorless oil. Yield: 372.0 mg, 53.0%. ^1^H NMR (400 MHz, CDCl_3_) δ 4.87 (m, 1H), 4.15 (t, *J* = 7.0 Hz, 4H), 2.49 (tt, *J* = 7.0, 2.4 Hz, 4H), 2.32 (m, 8H), 2.24 (s, 6H), 2.16 (tt, *J* = 6.9, 2.3 Hz, 4H), 1.81 (m, 2H), 1.63 (m, 4H), 1.45 (m, 12H), 1.30 (s, 16H), 0.92 (t, *J* = 7.2 Hz, 6H). MS (Water Acquity LC-MS): MW calc’d for C_43_H_76_NO_6_ (M + H^+^): 702.56, found: 702.63.

Synthesis of di(oct-3-yn-1-yl) 9-((4-(dimethylamino)butanoyl)oxy)heptadecanedioate (A4): The diacid 4 (0.43 g, 1 mmol) was dissolved in 20 mL of dichloromethane and 3-Octyn-1-ol (0.31 g, 2.44 mmol) was added to it followed by Hunig’s base (0.68 g, 4.9 mmol) and DMAP (12 mg). To this mixture EDCI (0.47 g, 2.44 mmol) was added and the reaction mixture was stirred at room temperature overnight. The reaction mixture was then diluted with CH_2_Cl_2_ (40 mL) and washed with saturated NaHCO_3_ (50 mL), water (60 mL), and brine (60 mL). The combined organic layers were dried over anhydrous Na_2_SO_4_ and solvents were removed in vacuo. The crude product thus obtained was purified by Combiflash Rf purification system (40 g silicagel, 0–10% MeOH in CH_2_Cl_2_) to afford the compound A4 as a colorless oil. Yield: 217.9 mg, 33.8%. ^1^H NMR (400 MHz, CDCl_3_) δ 4.87 (m, 1H), 4.15 (t, *J* = 7.0 Hz, 4H), 2.49 (tt, *J* = 7.0, 2.4 Hz, 4H), 2.32 (m, 8H), 2.24 (s, 6H), 2.16 (tt, *J* = 6.9, 2.3 Hz, 4H), 1.81 (m, 2H), 1.63 (m, 4H), 1.45 (m, 12H), 1.30 (s, 16H), 0.92 (t, *J* = 7.2 Hz, 6H). MS (Water Acquity LC-MS): MW calc’d for C_39_H_68_NO_6_ (M + H^+^): 646.50, found: 646.85.

Synthesis of di(non-3-yn-1-yl) 9-((4-(dimethylamino)butanoyl)oxy)heptadecanedioate (A5): The diacid 4 (0.43 g, 1 mmol) was dissolved in 20 mL of dichloromethane and 3-Nonyn-1-ol (0.34 g, 2.44 mmol) was added to it followed by Hunig’s base (0.68 g, 4.9 mmol) and DMAP (12 mg). To this mixture EDCI (0.47 g, 2.44 mmol) was added and the reaction mixture was stirred at room temperature overnight. The reaction mixture was then diluted with CH_2_Cl_2_ (40 mL) and washed with saturated NaHCO_3_ (50 mL), water (60 mL), and brine (60 mL). The combined organic layers were dried over anhydrous Na_2_SO_4_ and solvents were removed in vacuo. The crude product thus obtained was purified by Combiflash Rf purification system (40 g silicagel, 0–10% MeOH in CH_2_Cl_2_) to afford the compound A5 as a colorless oil. Yield: 112.2 mg, 16.7%. ^1^H NMR (400 MHz, CDCl_3_) δ 4.87 (m, 1H), 4.14 (t, *J* = 7.0 Hz, 4H), 2.49 (tt, *J* = 7.0, 2.4 Hz, 4H), 2.33 (dd, *J* = 15.6, 7.8 Hz, 8H), 2.25 (s, 6H), 2.15 (tt, *J* = 7.1, 2.4 Hz, 4H), 1.81 (m, 2H), 1.62 (dd, *J* = 14.4, 7.2 Hz, 4H), 1.48 (dd, *J* = 14.1, 7.0 Hz, 8H), 1.36 (m, 24H), 0.91 (dd, *J* = 9.3, 4.9 Hz, 6H). MS (Water Acquity LC-MS) (*m/z*): MW calc’d for C_41_H_72_NO_6_ (M + H^+^): 674.53, found: 674.26.

Synthesis of di(dec-3-yn-1-yl) 9-((4-(dimethylamino)butanoyl)oxy)heptadecanedioate (A6): The diacid 4 (0.43 g, 1 mmol) was dissolved in 20 mL of dichloromethane and 3-decyn-1-ol (0.38 g, 2.44 mmol) was added to it followed by Hunig’s base (0.68 g, 4.9 mmol) and DMAP (12 mg). To this mixture EDCI (0.47 g, 2.44 mmol) was added and the reaction mixture was stirred at room temperature overnight. The reaction mixture was then diluted with CH_2_Cl_2_ (40 mL) and washed with saturated NaHCO_3_ (50 mL), water (60 mL), and brine (60 mL). The combined organic layers were dried over anhydrous Na_2_SO_4_ and solvents were removed in vacuo. The crude product thus obtained was purified by Combiflash Rf purification system (40 g silicagel, 0–10% MeOH in CH_2_Cl_2_) to afford the compound A6 as a colorless oil. Yield: 208.4 mg, 29.7%. ^1^H NMR (400 MHz, CDCl_3_) δ 4.86 (m, 1H), 4.13 (t, *J* = 7.0 Hz, 4H), 2.48 (tt, *J* = 7.0, 2.3 Hz, 4H), 2.33 (m, 14H), 2.14 (tt, *J* = 7.1, 2.3 Hz, 4H), 1.83 (m, 2H), 1.61 (dd, *J* = 14.2, 7.1 Hz, 4H), 1.47 (m, 8 H), 1.35 (m, 28H), 0.89 (t, *J* = 6.9 Hz, 6H). MS (Water Acquity LC-MS) (*m/z*): MW calc’d for C_43_H_76_NO_6_ (M + H^+^): 702.56, found: 702.68.

### mRNA synthesis

mRNA was synthesized by Translate Bio^®^ using T7 RNA polymerase-mediated transcription from a linearized DNA template, which incorporates the 5ʹ and 3ʹ untranslated regions (UTRs), and a poly(A) tail (no nucleotide modifications). The final mRNA utilizes Cap1 to increase mRNA translation efficiency.

### Lipid nanoparticle synthesis

Lipid nanoparticles (LNPs) were prepared by mixing an ethanol phase containing lipids with mRNA in an aqueous phase in a microfluidic chip device^41^. In brief, the ethanol phase was prepared by solubilizing a mixture of ionizable lipid (i.e., A4, A5, or A6), 1,2-dioleoyl-sn-glycero-3-phosphoethanolamine (DOPE, Avanti), cholesterol (Sigma) and 1,2-dimyristoyl-sn-glycero-3-phosphoethanolamine-N-[methoxy(polyethylene glycol)-2000] (ammonium salt) (C14-PEG 2000, Avanti) at a predetermined molar ratio in ethanol. The aqueous phase was prepared in 10 mM citrate buffer (pH 3.5) with ether Luc mRNA (Firefly luciferase mRNA, Shire), or Cre mRNA (Cre-recombinase mRNA, TriLink). Syringe pumps were used to mix the aqueous and ethanol phases at a ratio of 3:1. The resulting LNPs were dialyzed against 1×PBS in a 20,000 MWCO cassette (Invitrogen) at 4 °C for 2 h. A modified Quanti-iI RiboGreeen RNA assay (Invitrogen) was used to calculate the nucleic acid encapsulation as previously described.

### In situ determination of pKa using TNS

The apparent pKa of each cationic lipid was determined using a fluorescent probe 2-(*p*-toluidino)-6-napthalene sulfonic acid (TNS) and preformed LNPs composed of cationic lipid/DOPE/cholesterol/PEG-lipid (35:16:35:2.5 mol%) in PBS at a concentration of ~6 mM total lipid^7^. In brief, TNS was prepared as a 100 μM stock solution in distilled water. LNPs were diluted to 100 μM of total lipids in 90 μL of buffered solutions (triplicates) containing 10 mM HEPES, 10 mM 4-morpholineethanesulfonic acid, 10 mM ammonium acetate, 130 mM NaCl, where the pH ranged from 2.71 to 11.5. Ten microliters of stock TNS was added to the LNP solutions and mixed well in a black 96-well plate. Fluorescence intensity was monitored in a Tecan Pro200 plate reader using excitation and emission wavelengths of 321 and 445 nm. With the resulting fluorescence values, a sigmoidal plot of fluorescence versus buffer pH was created. The log of the inflection point of this curve was the apparent pKa of the LNP formulation.

### Isolation of protein corona

All experiments were conducted four times to ensure reproducibility. LNP suspensions were incubated with an equal amount of mouse serum for 1 h at 37 °C (total volume of 350 μL). The serum coated LNPs were separated from free serum proteins by gel permeation chromatography on Sepharose CL-2B (Sigma) using phosphate buffered saline (pH 7.4) as the eluent. To confirm the separation of LNP and serum, mouse serum was labeled with Alexa Fluor^Tm^ 488 protein labeling kit (Invitrogen, Inc.) and purified according to manufacturer’s instructions. LNPs were labeled with Rhodamine-PE. The separation of LNPs (with corona) and proteins was analyzed by measuring fluorescence intensities in different flow fractionations (Fig. S6). Fractions containing LNPs (with protein corona) were merged and lyophilized for 2 days. Finally, the lyophilized powders were dissolved in cold acetone to remove lipid content and denatured with 8 M urea at 65 °C for 15 min, and processed for SDSPAGE gel or proteomic assays.

### Proteomic assay

For proteomic assays, the protein corona was isolated and purified using the gel permeation chromatography as described in the above section. Then the protein pellets were denatured using 8 M urea at 65 °C for 15 min and digested using trypsin (Promega, Inc.) according to the method developed by Shevchenko et al.^42^. The resulting peptide mixtures were re-suspended in 0.1% formic acid and analyzed by electrospray liquid chromatography mass spectrometry (LC-MS/MS) using an HPLC (Surveyor, ThermoFinnigan, CA) interfaced with an LTQ Orbitrap (ThermoFinnigan). The data have been analyzed against mouse protein databases in order to identify the mouse proteins in the corona on the nanoparticles. The identified proteins have been ordered according to their abundance and are shown in (Supplementary Table 1)^43^.

### Hemolysis

A hemolysis assay was performed according to a previous protocol^37^. In brief, Human RBCs (Innovative Research) were washed three times with 1×PBS and diluted in either citrate buffer saline (pH 5.5, 20 mM citrate buffer, 130 mM NaCl) or 1×PBS to a 4% vol/vol RBC solution. In a 96-round bottom plate, 100 μL blank LNPs formulated at an equivalent concentration of 0.5 mg mRNA/μL were added to 100 μL RBC solution in either CBS or PBS and incubated at 37 °C for 1 h (triplicates). After incubation, the plate was centrifuged at 4 °C at 1000 × *g* for 5 min. The whole plate was imaged using an ELISpot Imaging System. And then, the supernatant was transferred into a clear 96-well plate and UV absorption was read at 540 nm using Tecan Pro200 Plate Reader. Positive and negative controls were carried out with 0.1% Triton-X and buffer alone, respectively. To evaluate the interaction of protein on LNP fusion, serum protein, albumin, and ApoE were pre-incubated with particles for 15 min at determined concentration and transferred to 96-well plate co-cultured with RBC according to the above protocols.

### Membrane fusion assay

Membrane fusion assays were performed using a well-known method as described previously^38,44^ with the following modifications. Rhodamine-PE and NBD-PE dual labeled endosomal vesicles (42% DOPE, 17% DOPC, 13% LBPA, and 28% cholesterol) were prepared using a thin film method using citrate buffer as a hydration solution. Particles were sonicated and protruded through 100 μm membrane and added to a black 96-well plate at 30 µL per well. Non-fluorescent labeled cKKE12, A6 or Syn-3 LNPs were separately prepared using a microfluidic device as mentioned above and diluted using citrate buffer. Upon mixing an equal volume of LNPs with the donor vesicles, NBD fluorescence was monitored at 540 nm with excitation at 465 nm. Non-fluorescence vesicles and fluorescent LNPs were set as controls, respectively. The lipid mixing kinetic traces were fitted to a single exponential function using Graphpad Prism 7.0. To evaluate fit, we generated an R2 value using the experimental data and calculated the value obtained from fitting the curve.

### LNP subcellular distribution and subcellular fractionation

hEPO mRNA containing LNPs were incubated with primary hepatocytes (with 10% serum) on ice for 30 min to ensure complete binding. Then, cells were washed extensively with cold PBS four times and transferred to 37 °C incubator. At predetermined time points, cells were scraped from the plates and subjected to subcellular fractionation using subcellular fractionation kit (Thermo) according to the manufacturer’s protocols. Cytoplasmic mRNAs and proteins were isolated using CPB buffer. mRNA was further extracted and purified using an RNeasy kit (Qiagen) and reverse transcribed into cDNA. The amount of hEPO mRNA was then quantified using cell cycler realtime PCR (rt-PCR) with TaqMan® Assay primers (hEPO: Hs01071097_m1, Thermo, Inc., predesigned primer). To compare the mRNA localized in cytoplasm and in the membrane organelles, mRNA and proteins from cytoplasm and organelles were isolated 1.5 h after incubating with LNPs following the manufacturer’s protocol. To confirm the successful separation of cytoplasm and organelles, lysosome associated membrane protein LAMP-1 was evaluated by western blotting. The presence and absence of LAMP-1 in organelle and cell cytoplasm demonstrates the successful separation. mRNA was then purified and quantified using rt-PCR.

### In vitro mRNA uptake and transfection efficiency assay

Mouse primary hepatocytes were isolated using a standard two-step collagenase perfusion technique^45^ and cultured on collagen coated plates (BD Biosciences, Franklin Lakes, NJ). HeLa cells were obtained from ATCC (Manassas, VA). Cells were maintained in Dulbecco’s modified Eagle’s medium (Invitrogen, Carlsbad, CA) supplemented with penicillin, streptomycin, and fetal bovine serum (Gibco Laboratories, Gaithersburg, MD). For transfection experiments, cells were seeded in 96-well plates (Greiner, Kremsmünster, Austria) overnight and then Fluc mRNA containing LNP formulations were incubated with cells overnight. The luciferase expression efficiency and cytotoxicity were measured by One-Glo^TM^ + Tox luciferase Reporter and Cell Viability assay kit (Promega, Madison, WI) following instructions. In protein pre-association experiments, we used a similar method previously reported by Akinc et al.^24^. LNPs were pre-associated with human recombinant apoE3 (Fitzgerald Industries, Acton, MA), human serum albumin (Sigma) or fetal bovine serum (Gibco) at predetermined concentrations for 15 min at 37 °C before adding the same protein cultured medium containing LNPs to cells. We added 0.5–6 mg/mL serum albumin, and 0.2–3 μg/mL of ApoE, and 10% serum to media to ensure that serum albumin/ApoE/serum ratios were similar to ratios found in physiological conditions^46^. Cells were analyzed 24 h later using the same assay described above. To study cellular uptake, cells were incubated with Cy5-labeled Fluc mRNA (TriLink, San Diego, CA) or 0.1 mol% Rhodamine-PE (Avanti 810150, Alabaster, AL) labeled LNPs for different time points, fixed in 4% paraformaldehyde and then quantified using flow cytometry (LSRII, BD) with a high-throughput setting (HTS-FACs). For the endocytosis pathway knockout assay, siRNAs (CLTC siRNA (NM_001003908), AM16708 from Thermo; RAC-1 siRNA (NM_009007), AM164723 from Thermo; Cdc42 siRNA (NM_009861), SASI_Mm02_00317284, SASI_Mm02_00317285 from Sigma; CAV1 siRNA (NM_007616), SASI_Mm01_00141141, SASI_Mm01_00141142 from Sigma; LDLR siRNA (NM_010700), SASI_Mm01_00289059, SASI_Mm01_00289058 from Sigma) against different pathway-associated proteins were incubated with primary hepatocytes for 2 days before treated with LNPs. For inhibitor assays, fucoidan (Sigma) was co-incubated with LNPs at predetermined concentrations before tested for transfection efficiency and cellular uptake.

### Confocal microscopy

Intracellular release of mRNA from LNPs was visualized using confocal microscopy (Nikon). Cy5-labeled mRNA containing LNPs were pre-incubated with primary hepatocytes on ice for 30 min. After extensive washing (4 times with cold PBS), cells were transferred to 37 °C incubator. Cells were then washed and fixed at 5, 15, 30, and 60 min after incubation and then imaged using confocal microscope. To aid visualization, we adopted a similar method to Wittrup et al.^31^ to enhance weak signals (i.e., cytosolic mRNA) using modified display lookup tables (LUT). Briefly, we adjusted the LUT to minimize the fluorescence of aggregates in the endo-lysosomes and display fluorescence in the cytoplasm. To quantify the co-localization of Cy5-mRNA with lysotracker, we used Image J to modify the color threshold to select the co-localized pixels and quantify the ratio of co-localized pixels in the overall Cy5-mRNA pixels. Cy5-mRNA disseminated in cytoplasm were also quantified using Image J (through the “analyze particle size” module). Hepatocytes without LNP treatments were used as a control for the cytoplasm intensity adjustments (Supplementary Fig. 27). Florescence intensity was further quantified using Image J.

### Animal experiments

All experimental procedures were ethically approved and performed under the guidelines of the Division of Comparative Medicine by Massachusetts Institute of Technology. For in vivo luciferase expression assays, LNPs composed of various molar ratio of cKK-E12 and A6 were mixed and dialyzed before injection into mice. 5–15 µg Fluc mRNA per mouse was injected for each lipid group. Six hours after injection, mice were imaged using a bioluminescence approach in an IVIS kinetic imaging system (Perkin Elmer). Six-week-old male C57BL/6 mice was (Charles River) were used for the adenine-induced renal anemia model. Renal injury was induced by oral gavage with adenine (50 mg/kg bodyweight in 0.5% methylcellulose) daily for 4 weeks. The presence of renal anemia was confirmed by measuring hematocrit and hemoglobin levels and red blood cell count. One week after the end of adenine treatment, the following six mouse groups were prepared: group 1, no adenine treatment (control; *n* = 5); group 2, hEPO protein (0.03 mg/kg) (*n* = 8); group 3, hEPO mRNA containing cKK-E12/A6 LNPs (0.015 mg/kg) injected every 3 days after adenine treatment (*n* = 8); group 4, hEPO mRNA containing cKK-E12/A6 LNPs (0.03 mg/kg) injected weekly after adenine treatment (*n* = 8); group 5, hEPO mRNA containing cKK-E12 LNPs (0.015 mg/kg) injected every 3 days after adenine treatment (*n* = 8); group 6, saline injection after adenine treatment (*n* = 8). Blood samples were collected to measure the hematocrit and hemoglobin levels and EPO protein concentrations. The hematocrit levels were determined in blood samples withdrawn into glass capillary tubes. The hemoglobin levels were measured using ELISA (Abcam), and the numbers of red blood cells, white blood cells, and platelets were counted using a Coulter Counter Multisizer 3 (Beckman Coulter).

### Blood collection and analysis for hEPO

Blood was collected from mice via the tail vein, allowed to clot at room temperature in serum separator tubes (Fisher Scientific, Boston, MA). The tubes were then centrifuged at 2000 × *g* for 7 min and the sera samples aliquoted and stored at −80 °C until analysis. hEPO concentrations were determined using a hEPO ELISA assay (R&D Technologies, Cambridge, MA) according to the manufacturer’s instructions.

### Molecular dynamics (MD) simulation

MD simulations used all-atomic OPLS force fields with explicit water^47^. The TIP4P water model was used to improve dipole moment and structural properties^48^. Minor modifications on the hydrocarbon chains of ionizable lipids and DOPE were adopted from Nesterenko et al. to better correspond to the generally accepted Berger potential^49^. Customized parameters were taken from literature to adequately portray the chemical identity of the amine groups in A6 and cKK-E12 lipids^50^. Alkyne parameters in A6 are taken from the original OPLS force fields^47^. Topology and partial charges of A6, cKK-E12, and LBPA are given in Supplemental Materials and Methods (Supplementary Fig. 9–11). The time step of all simulation is 2 fs. Particle Mesh Ewald is used with a grid spacing of 0.16 nm for fast Fourier transformation^51^. Periodic boundary condition is applied in all three dimensions. The covalent bond in the lipid are constrained by LINCS whereas bonds in TIP4P water are constrained by SETTLE. Temperature is control by v-rescale whereas pressure is maintained at 1 atm. by Berendsen barostat. The cutoff distance of Van der Waal force and short-ranged columbic interaction is 1.0 nm. All MD simulation is performed using GROMACS 5.0.5^52^.

### Lipid membrane simulation

Lipid membrane is constructed by aligning the ionizable lipid, DOPE, and cholesterol randomly on a two-dimensional grid with a predefined ratio for each formulation. Two layers of water molecules were then added on top of the aligned bilayer. 150 mM NaCl^−^ with counterions are generated and added to the water layer to neutralize the system (Supplementary Fig. 12). The solvated membrane is equilibrated in the NVT ensemble for 20 ns and then simulated in NPT ensemble to condense the system. The final membrane system has 70 ionizable lipids, 38 DOPE, 92 cholesterol, and approximately 16k water molecules. Production run of 100 ns is performed. Tail Protrusion was calculated by measuring tail atoms that are 0.1 nm beyond the average height of phosphorus atoms of DOPE using the data collected from last 20 ns. Four carbon atoms in each tail end (for both A6 and cKK-E12) are counted for measurement. Lateral Diffusion coefficient is calculated from the mean square displacement by Einstein relation using the data collected from the last 50 ns of simulation.

### Vesicle fusion simulations

Vesicle synthesis was modified from the method described by Knecht and Marrink^53^. Instead of obtaining the lipid distribution by self-assembling the vesicle using a coarse-grained model, we constructed vesicles directly from the atomistic model to maintain a fixed composition of lipids in each leaflet. The amount of ionizable lipids in inner and outer layer of the vesicles are calculated based on the spherical area of each leaflet and their area per lipid in relaxed bilayer membranes. For A6 vesicles, the outer layer has 324 A6 lipids, 161 DOPE, and 161 cholesterol, whereas the inner layer has 160 A6 lipid, 80 DOPE, and 80 cholesterol. For cKK-E12 vesicles, the outer layer has 207 cKK-E12 lipids, 103 DOPE, and 103 cholesterol, whereas the inner layer has 106 cKK-E12 lipid, 53 DOPE, and 53 cholesterol. For endosomal vesicle, the outer layer has 274 DOPC, 109 DOPE, 83 LBPA, and 180 cholesterol, whereas the inner layer has 136 DOPC, 54 DOPE, 41 LBPA, and 89 cholesterol (Supplementary Table 2). The initial vesicle system is obtained using a three-step condensation procedure. First, the ionizable lipid, DOPE and cholesterol with a predefined ratio were aligned outwardly on a spherical surface to form the outer layer of the vesicle. The aligned outer layer was put in a large cubic water box. Water molecules inside the vesicle were removed. The water shell and outer layer were equilibrated in a canonical NVT ensemble for 20 ns. Then, the inner layer of the vesicle is prepared by aligning lipids inwardly on a spherical surface. The inner layer was added a water core with 5 nm diameter and equilibrated for 20 ns. Finally, the outer layer and the inner layer were combined (with a vacuum gap between the two layers) and equilibrated for 40 ns in a NPT (isothermal-isobaric) ensemble. The system was then condensed as the vacuum gap was eliminated, and a complete vesicle was formed with an average diameter of 14.3 nm in all three dimensions (Supplementary Fig. 18). To perform fusion simulation, two vesicle systems are combined into a large box with a dimension of 16 × 16 × 32 nm^3^ and ~60,000 water molecules (totaling ~1 M atoms). The system is then equilibrated for 10 ns. To induce fusion, the water layer (3-nm thickness) between the A6/cKK-E12 vesicles and endosomal vesicle is removed to enable direct contact of the two vesicles (Supplementary Fig. 20). Fusion simulation is performed in a NPT ensemble with fixed dimensions in *xy* and a freely adjustable dimension in *z*. Temperature is set to 350 K to accelerate the fusion process in a 50 ns production run^53^.

### Free energy calculation

Potential of mean force (PMF) is calculated to estimate free energy change along the reaction coordinate for lipid sprout and flip-flop. Umbrella sampling is used to constrain the lipid along the coordinate. The biasing potential acts on the amine group of the ionizable lipid with a force constant of 5000 kJ mol^−1^ nm^−2^ to keep a designated distance between the constrained group and the center of mass of the membrane. The interval of umbrella sampling is 0.1 nm and the total length of reaction coordinate is 5 nm (from membrane center to bulk water), which results in 50 sampling window for each ionizable lipids. The initial configuration of each sampling window is created by pulling the lipid to its constrained position by a week potential (500 kJ mol^−1^ nm^−2^). Each window is first equilibrated for 10 ns with the full biasing potential and sampled for 40 ns. The dimensions of the system for umbrella sampling is 5 × 5 × 18 nm^3^. The PMF profile is calculated from the force distribution of the constrained amines by the weighted histogram analysis method with a tolerance of 10^−4 ^^54^.

### Statistical analysis

Means (± standard deviation (SD)) were compared using one-way ANOVA with post-Dunnett tests for multiple comparisons. Student’s T-test was used to compare between two groups. For all tests, two-tailed *p* values <0.05 were considered statistically significant, and are shown in the figures as *p < 0.05, **p < 0.005, or ***p < 0.001. Prism 7.03 was used.

### Reporting summary

Further information on research design is available in the Nature Research Reporting Summary linked to this article.

## Supplementary information

Supplementary Information

Reporting Summary

## Data Availability

The data that support the findings of this study are available from the authors on reasonable request.
