# Supplementary material for: Synergistic lipid compositions for albumin receptor mediated delivery of mRNA to the liver
Source: Nat Commun. 2020 May 15;11:2424. doi: 10.1038/s41467-020-16248-y (PMC7229004; doi:10.1038/s41467-020-16248-y)
Supplement: Supplementary file 1 — Supplementary Information [file 41467_2020_16248_MOESM1_ESM.docx]

**Supplementary Materials for**

**Synergistic Lipid Compositions for Albumin Receptor Mediated, Delivery of mRNA to the Liver**

Lei Miao,^1†^ Jiaqi Lin,^1†^ Yuxuan Huang,^1^ Linxian Li,^1,2^ Derfogail Delcassian,^1,3,4^ Yifan Ge,^5,6^ Yunhua Shi,^1^ Daniel G. Anderson^1,3,7,8,9*^

^1^Koch Institute for Integrative Cancer Research, Massachusetts Institute of Technology, Cambridge, MA, 02142, USA

^2^Ming Wai Lau Centre for Reparative Medicine, Karolinska Institutet, Stockholm, 17177, Sweden

^3^Department of Anesthesiology, Boston Children’s Hospital, 300 Longwood Ave, Boston, MA 02115, USA

^4^Division of Regenerative Medicine and Cellular Therapy, University of Nottingham, Nottingham, NG7 2RD, UK

^5^Department of molecular biology, Massachusetts General Hospital, Cambridge, MA, 02114, USA

^6^Department of genetics, Harvard Medical School, Cambridge, MA, 02115, USA

^7^Department of Chemical Engineering, Massachusetts Institute of Technology, Cambridge, MA, 02142, USA

^8^Institute for Medical Engineering and Science, Massachusetts Institute of Technology, Cambridge, MA, 02139, USA

^9^Harvard-MIT Division of Health Science and Technology, Massachusetts Institute of Technology, Cambridge, MA, 02139, USA

^†^These authors contributed equally to this work.

^*^Corresponding author. Email: [dgander@mit.edu](mailto:dgander@mit.edu) (D.G.A.)

**Supplementary Tables**

**Supplementary Table 1： % of total protein coated on LNPs (analyzed by proteomics)**

| Category | Protein Name | cKK-E12 LNP | | | | A6 LNP | | | | Syn-3 LNP | | | |
| --- | --- | --- | --- | --- | --- | --- | --- | --- | --- | --- | --- | --- | --- |
| Immunoglobin | Ig kappa chain V-V region K2 | 0.180 | 0.500 | 0.171 | 0.310 | 0.592 | 0.477 | 0.366 | 0.474 | 0.189 | 0.552 | 0.272 | 0.352 |
|  | Ig heavy chain V region | 0.218 | 0.957 | 0.127 | 0.501 | 1.142 | 0.930 | 0.620 | 0.905 | 0.110 | 1.055 | 0.239 | 0.550 |
|  | Ig kappa chain V-II region | 0.294 | 0.803 | 0.316 | 0.582 | 1.192 | 0.811 | 0.740 | 0.916 | 0.344 | 0.647 | 0.394 | 0.487 |
|  | Ig gamma-2A chain C region | 0.735 | 1.702 | 0.596 | 1.273 | 1.580 | 2.123 | 1.257 | 1.690 | 0.844 | 1.931 | 1.225 | 1.438 |
|  | Ig gamma-2B chain C region | 0.670 | 1.807 | 0.661 | 1.287 | 1.332 | 0.966 | 1.149 | 1.125 | 0.794 | 2.319 | 0.937 | 1.495 |
|  | Ig gamma-3 chain C region | 0.710 | 1.890 | 0.957 | 1.227 | 1.186 | 1.006 | 1.194 | 1.117 | 0.848 | 1.345 | 1.023 | 1.119 |
|  | Immunoglobulin J chain | 0.943 | 2.064 | 0.793 | 1.516 | 3.490 | 1.678 | 1.998 | 2.411 | 0.888 | 1.905 | 0.930 | 1.351 |
|  | Ig kappa chain C region | 1.658 | 3.117 | 1.377 | 2.393 | 4.921 | 4.021 | 3.441 | 4.156 | 1.501 | 4.348 | 2.334 | 2.991 |
|  | Ig gamma-1 chain C region | 1.523 | 3.663 | 2.792 | 2.753 | 3.003 | 3.130 | 2.035 | 2.804 | 1.336 | 5.152 | 2.400 | 3.328 |
|  | Ig mu chain C region | 5.587 | 3.584 | 4.595 | 4.560 | 5.960 | 7.601 | 9.485 | 7.473 | 5.111 | 3.767 | 4.389 | 4.368 |
| Lipoprotein | Apolipoprotein A-II | 0.107 | 0.403 | 0.293 | 0.320 | 0.509 | 0.538 | 0.491 | 0.519 | 0.385 | 0.395 | 0.272 | 0.372 |
|  | Apolipoprotein A-I | 1.151 | 2.700 | 2.740 | 2.377 | 3.317 | 3.551 | 3.118 | 3.386 | 3.553 | 2.391 | 3.361 | 3.031 |
|  | Apolipoprotein A-IV | 1.642 | 2.404 | 5.171 | 2.833 | 3.660 | 4.988 | 3.229 | 3.865 | 2.162 | 1.937 | 1.892 | 1.991 |
|  | Apolipoprotein B | 3.151 | 3.600 | 8.560 | 4.681 | 4.939 | 4.974 | 4.831 | 5.125 | 5.004 | 3.378 | 4.337 | 4.143 |
|  | Apolipoprotein E | 9.135 | 8.191 | 11.915 | 10.215 | 11.396 | 10.834 | 10.723 | 10.986 | 11.630 | 8.798 | 10.247 | 9.175 |
| Complement pathway | Complement C1r-A | 0.145 | 0.271 | 0.289 | 0.268 | 0.623 | 0.284 | 0.343 | 0.425 | 0.158 | 0.170 | 0.130 | 0.166 |
|  | Complement C4 | 0.298 | 0.757 | 0.491 | 0.439 | 0.636 | 0.527 | 0.452 | 0.542 | 0.291 | 0.706 | 0.388 | 0.469 |
|  | Complement C1q | 0.776 | 1.139 | 0.981 | 0.967 | 2.824 | 2.631 | 2.204 | 2.595 | 0.548 | 0.664 | 0.367 | 0.550 |
|  | Complement C3 | 9.198 | 6.264 | 8.052 | 7.243 | 5.083 | 6.123 | 5.639 | 5.158 | 7.999 | 7.408 | 5.927 | 7.185 |
| Acute phase protein | Plasminogen | 0.168 | 0.416 | 0.146 | 0.359 | 0.203 | 0.248 | 0.206 | 0.211 | 0.148 | 0.411 | 0.221 | 0.280 |
|  | Alpha-1-antitrypsin 1 | 0.403 | 0.717 | 0.546 | 0.554 | 0.579 | 0.581 | 0.616 | 0.609 | 0.562 | 0.996 | 0.580 | 0.760 |
|  | Alpha-1-antitrypsin 2 | 0.417 | 0.818 | 0.317 | 0.615 | 0.579 | 1.127 | 0.532 | 0.771 | 0.387 | 2.199 | 0.792 | 1.286 |
|  | Alpha-2-HS-glycoprotein | 0.728 | 1.980 | 0.840 | 1.286 | 0.931 | 0.992 | 0.867 | 0.937 | 0.923 | 1.385 | 1.061 | 1.156 |
|  | NF-kappa-B inhibitor zeta | 1.429 | 3.437 | 2.619 | 2.537 | 2.817 | 2.845 | 1.909 | 2.630 | 1.253 | 4.969 | 2.252 | 3.121 |
| Tissue Leakeage | Keratin, type I cytoskeletal 16 | 0.482 | 0.686 | 0.410 | 0.638 | 0.488 | 0.522 | 0.704 | 0.573 | 0.479 | 0.443 | 0.418 | 0.451 |
|  | Keratin, type II cytoskeletal 79 | 0.522 | 0.706 | 0.303 | 0.620 | 0.485 | 0.503 | 0.969 | 0.636 | 0.381 | 0.354 | 0.410 | 0.349 |
|  | Keratin, type II cytoskeletal 2 | 0.503 | 0.823 | 0.265 | 0.568 | 0.562 | 0.466 | 0.886 | 0.608 | 0.300 | 0.354 | 0.478 | 0.372 |
|  | Keratin, type I cytoskeletal 13 | 0.616 | 0.988 | 0.601 | 0.719 | 0.855 | 0.736 | 1.079 | 0.873 | 0.525 | 0.594 | 1.021 | 0.702 |
|  | Keratin, type I cytoskeletal 14 | 0.738 | 1.309 | 0.692 | 0.973 | 1.205 | 1.004 | 1.531 | 1.240 | 0.702 | 0.970 | 1.095 | 0.928 |
|  | Keratin, type I cytoskeletal 42 | 0.983 | 2.035 | 0.476 | 1.413 | 1.032 | 0.932 | 1.397 | 1.121 | 0.487 | 0.967 | 0.596 | 0.725 |
|  | Keratin, type II cytoskeletal 6A | 1.644 | 2.737 | 0.692 | 1.893 | 0.642 | 0.622 | 2.105 | 1.106 | 0.514 | 0.503 | 0.834 | 0.611 |
|  | Keratin, type II cytoskeletal 1 | 1.914 | 2.695 | 1.211 | 2.068 | 2.393 | 1.685 | 3.434 | 2.431 | 1.184 | 1.125 | 2.080 | 1.411 |
|  | Keratin, type II cytoskeletal 5 | 2.141 | 4.053 | 1.561 | 2.804 | 2.866 | 2.230 | 3.436 | 2.844 | 1.584 | 1.867 | 2.103 | 1.878 |
|  | Keratin, type I cytoskeletal 10 | 6.580 | 4.793 | 2.671 | 4.782 | 2.728 | 2.771 | 3.974 | 3.151 | 2.376 | 2.766 | 2.616 | 2.763 |
| Coagulation | Thrombospondin-1 | 0.223 | 0.507 | 0.193 | 0.330 | 0.503 | 0.361 | 0.422 | 0.423 | 0.207 | 0.625 | 0.250 | 0.372 |
|  | Ficolin-1 | 0.369 | 0.484 | 0.254 | 0.390 | 1.097 | 0.653 | 0.636 | 0.817 | 0.271 | 0.328 | 0.645 | 0.406 |
|  | Gelsolin | 0.366 | 0.573 | 0.468 | 0.482 | 0.413 | 0.525 | 0.430 | 0.453 | 1.169 | 0.338 | 0.407 | 0.598 |
|  | Fibronectin = | 0.476 | 1.463 | 0.633 | 0.945 | 1.095 | 0.885 | 0.982 | 1.002 | 0.566 | 0.991 | 0.707 | 0.802 |
|  | Kininogen-1 | 3.340 | 2.138 | 2.619 | 2.498 | 2.817 | 2.936 | 1.909 | 2.630 | 3.661 | 2.550 | 2.252 | 3.121 |
| Other Component | Transferrin receptor protein 1 | 0.376 | 0.339 | 0.258 | 0.325 | 1.144 | 0.874 | 0.841 | 0.920 | 0.303 | 0.392 | 0.296 | 0.352 |
|  | Liver carboxylesterase 1 | 0.318 | 0.674 | 0.240 | 0.449 | 0.377 | 0.870 | 0.407 | 0.567 | 0.295 | 0.514 | 0.792 | 0.542 |
|  | Serine protease inhibitor A3K | 0.317 | 0.697 | 0.365 | 0.498 | 0.554 | 0.723 | 0.498 | 0.608 | 0.422 | 0.884 | 1.074 | 0.815 |
|  | Vitronectin OS=Mus musculus | 0.358 | 0.847 | 0.377 | 0.578 | 0.622 | 0.547 | 0.497 | 0.561 | 0.447 | 0.447 | 0.395 | 0.437 |
|  | Transthyretin OS=Mus musculus | 0.501 | 1.327 | 0.382 | 0.828 | 1.003 | 1.652 | 0.729 | 1.165 | 0.399 | 1.431 | 1.303 | 1.018 |
|  | Serotransferrin OS=Mus musculus | 14.578 | 7.352 | 12.879 | 10.836 | 10.792 | 11.073 | 10.603 | 10.968 | 15.303 | 9.792 | 11.970 | 11.891 |
|  | Serum albumin OS=Mus musculus | 21.388 | 9.590 | 17.106 | 14.267 | 3.836 | 4.446 | 5.086 | 4.475 | 21.458 | 12.937 | 22.286 | 18.291 |

**Supplementary Table 2. Simulation details.**

| **System** | **Composition^1^** | **Size (nm)** | **Length (ns)** | **Times** |
| --- | --- | --- | --- | --- |
| A6 bilayer | A6, DOPE, cholesterol | 6.6×6.6×15.6 | 120 | 3 |
| cKK-E12 bilayer | cKK-E12, DOPE, cholesterol | 7.5×7.5×12.3 | 120 | 3 |
| Syn-3 bilayer | A6, cKK-E12, DOPE, cholesterol | 7.3×7.3×13.0 | 120 | 3 |
| A6 PMF^2^ | A6, DOPE, cholesterol | 5.3×5.3×16.0 | 10 | 100^3^ |
| cKK-E12 PMF^2^ | cKK-E12, DOPE, cholesterol | 6.4×6.4×9.1 | 10 | 100^3^ |
| A6 vesicle | A6, DOPE, cholesterol | 15.2×15.2×13.4 | 20 (350K) | 3 |
| cKK-E12 vesicle | cKK-E12, DOPE, cholesterol | 14.9×14.9×15.8 | 20 (350K) | 3 |
| Endosomal vesicle | LBPA, DOPC, DOPE, cholesterol | 15.3×15.3×15.2 | 20 (350K) | 1 |
| A6+endosomal fusion | \ | 16.0×16.0×26.0 | 42 (350K) | 1 |
| cKK-E12+endosomal fusion | \ | 15.0×15.0×27.6 | 42 (350K) | 1 |

^1^The ratio between A6 and DOPE; cKK-E12 and DOPE in the mono bilayer were kept at 2:1 (molar ratio), which are same to both the *in vivo* and *in vitro* transfection formulations. 23.5 w/w% Cholesterol was used in the model. C14-PEG2000 was removed from the bilayer to simplify the bilayer structure. For endosomal vesicle, the molar ratio of LBPA, DOPC, DOPE, cholesterol is 13%, 42%, 17%, 28%.

^2^Potential of mean force (PMF)

^3^Number of umbrella sampling windows in PMF calculation.

**Supplementary Figures**

**Supplementary Figure 1. The natural metabolites of linoleic acid.**

**Supplementary Figure 2. Intravenous injection of hEPO mRNA loaded mono lipid LNPs.** hEPO expression was measured 6 h after injection (0.75 mg/kg hEPO mRNA). Formulation composed of ionizable lipid, DOPE, Cholesterol and PEG14-2000. Of note, this DOPE formulation has optimal mRNA delivery efficiency for cKK-E12 and C12-200, but slightly lower mRNA delivery efficiency for MC3 as compared to the traditional DSPC formulation. The delivery efficiency of alkyne biodegradable lipidoids (A1,2,5,6) are higher than L319 but slightly lower than MC3. Data are presented as mean±SD.


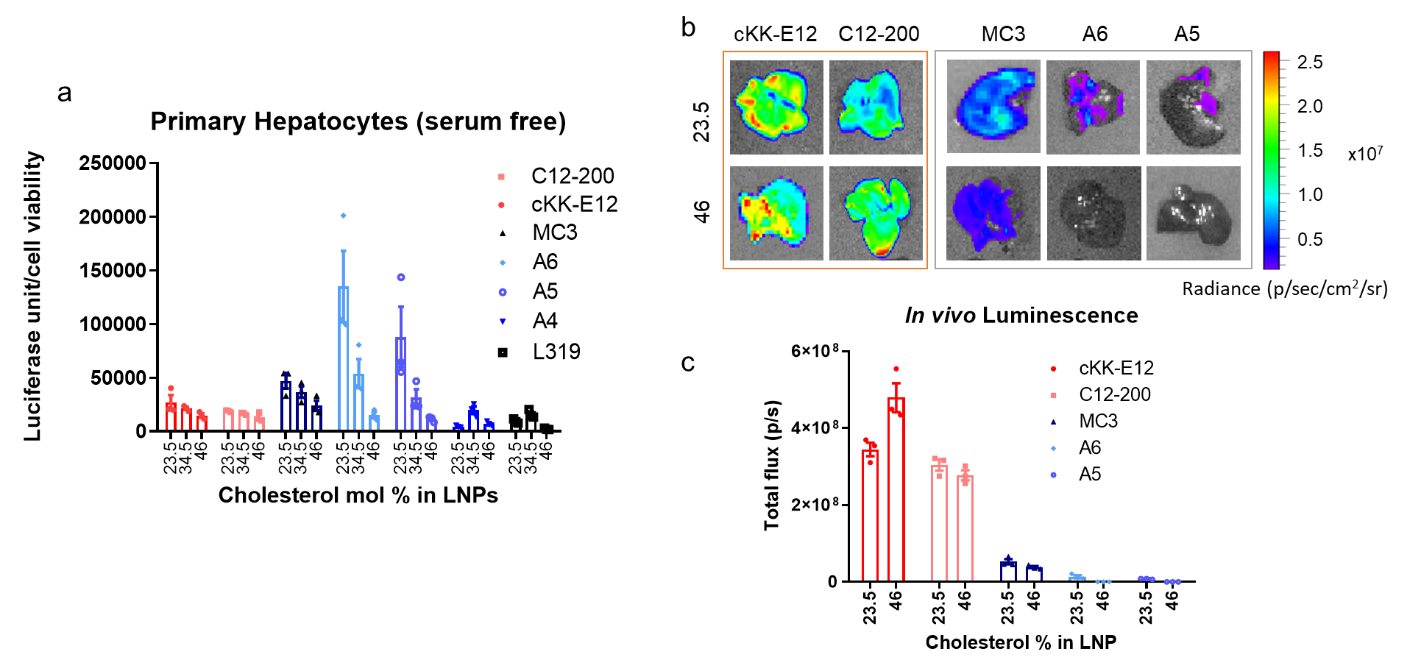


**Supplementary Figure 3. The *in vivo* and *in vitro* FLuc mRNA delivery efficacy of different LNPs were compared at different formulations.** In the 3 formulations tested, the ratio of ionizable lipid/DOPE was fixed at 2:1 (*mol:mol*), while the cholesterol amount in the formulation was varied (from 23.5 to 46 mol%). Luciferase activity was first tested *in vitro* on primary hepatocytes 24 h after incubation in serum free medium at mRNA concentration of 0.1 mg/well, in 96 well plates (a). mRNA delivery efficiency was also compared *in vivo* 6h after intravenous injection of Fluc mRNA LNPs (0.75 mg/kg). Results suggested that alkyne lipidoids presented different *in vivo* and *in vitro* mRNA delivery efficacy. Data are presented as mean±SD.

*
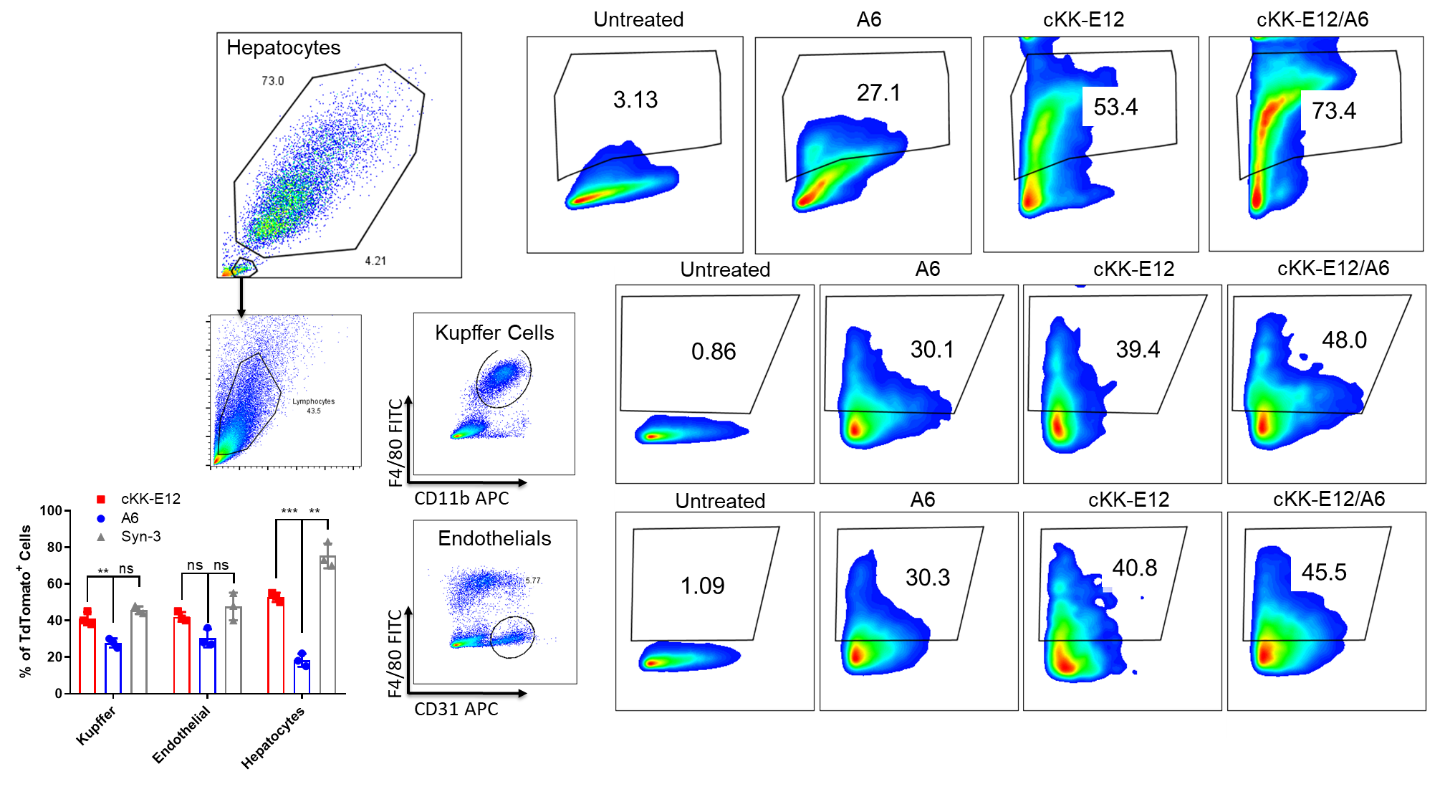
*

**Supplementary Figure 4. tdTomato expression in the hepatocytes and non-parenchymal cells (NPCs) 2 days after intravenous injection of Cre mRNA loaded A6, cKK-E12 and Syn-3 LNPs (0.75 mg/kg Cre mRNA).** Cre mRNA can be delivered and expressed in both hepatocytes and NPCs. However, hepatocytes are the major cells that express the protein. Also, results suggest that Syn-3 LNPs significantly increases the % and mean fluorescence intensity of hepatocytes that express the protein. Data are presented as mean±SD. **, *P* < 0.01; ***, *P* < 0.001, ns, no significant difference, One-way ANOVA.

*
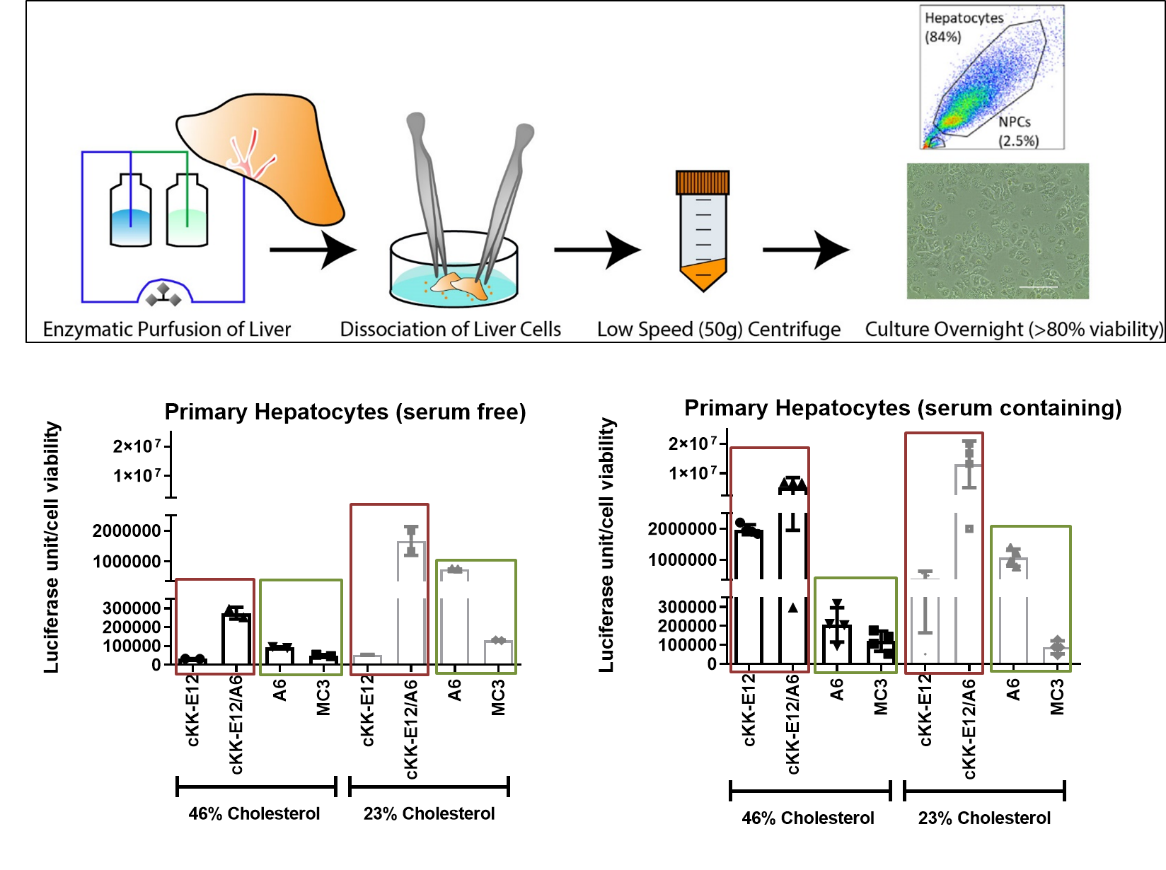
*

**Supplementary Figure 5. Culturing in primary hepatocytes in or absence of serum demonstrates the role of serum proteins in mRNA delivery by cKK-E12 containing LNPs.** Schematic on top shows the isolation process and purity of primary hepatocytes. Data are presented as mean±SD.


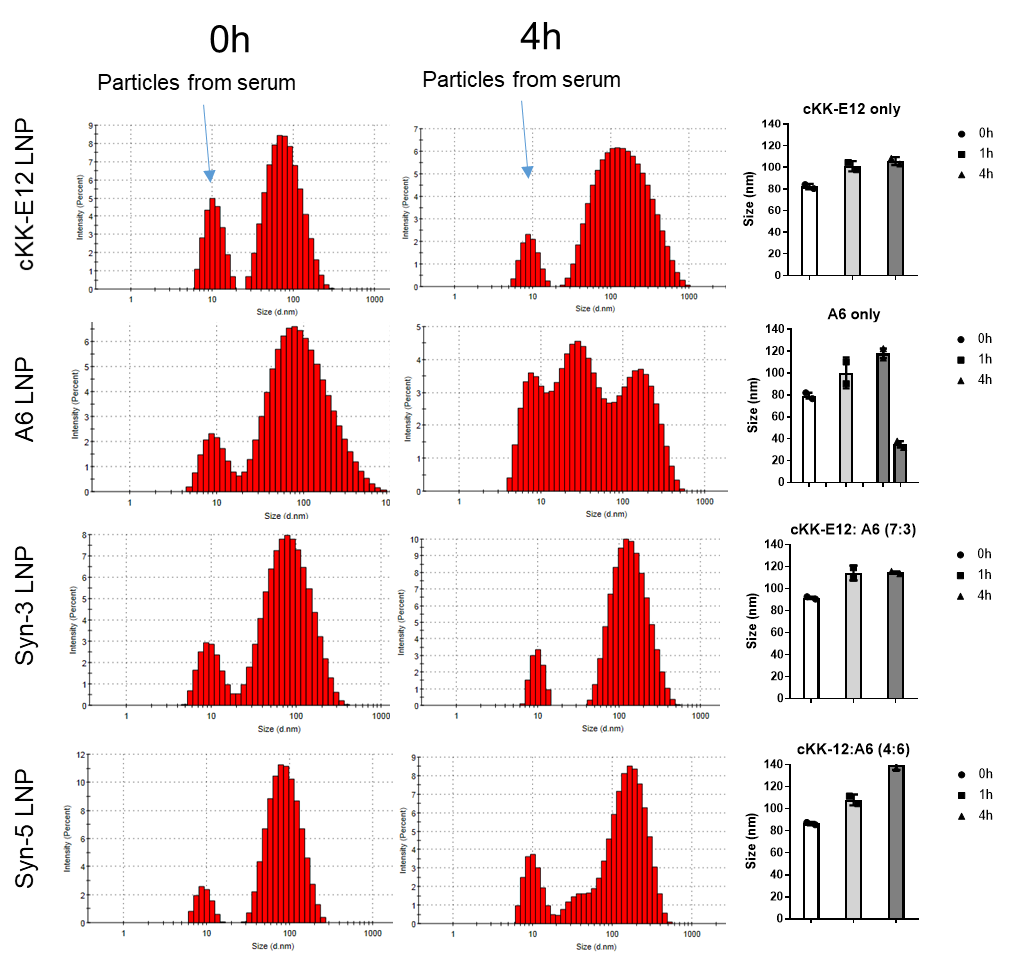


**Supplementary Figure 6. Particle size distribution of LNPs incubated in 10% serum PBS medium.** cKK-E12, A6 and Syn-LNPs were incubated with 10% mouse serum in phosphate buffered saline (PBS ) at 37°C for 4h (shaking at 50g). Changes in particle size were measured using Dynamic Light Scattering (DLS). Results suggest that cKK-E12 and Syn-3 LNPs were stable within serum for at least 4h. Slightly increased particle size was observed for these two LNPs after incubation with serum due to the forming of protein corona, however, no aggregation and decomposition were observed. In contrast, A6 and Syn-5 LNPs started to decompose at 4h, which may due to the degradation of biodegradable alkyne lipids. Data are presented as mean±SD.

**Supplementary Figure 7. Chromatography separation of LNPs (coated with protein corona) and free unbound serum proteins.** Fluor^TM^ 488 labeled serum protein (mouse) was incubated with Rhodamine-PE labeled LNPs for 1h at 37°C. Then the serum coated LNPs were separated from free unbound serum proteins by gel permeation chromatography. LNP protein mixtures were loaded on the column, and diluent fractions were collected and analyzed by fluorescent reading. Rectangle highlights the fractions contained both Fuor^TM^ 488 labeled serum protein and Rhodamine-PE labeled LNPs. Since no fluorescence was observed in the serum only sample in the same rectangle fractions (~fraction 1-30) as compared to LNP protein mixture samples, we identified this group of proteins as protein corona that were coated onto LNPs. These fractions were collected, combined and used for proteomic assay. Of note, after isolation and combined of the fractions, concentration of LNPs were measured. The same amount of LNPs (containing different amount of corona proteins) in each group were processed for proteomic analysis.

**Supplementary Figure 8.** **Hepatocyte uptake of Cy5-mRNA encapsulated LNPs in the presence or absence of albumin** (6 mg/mL albumin, n = 4/group). Results suggest that albumin aids the cellular uptake of cKKE-12 containing LNPs. Data are presented as mean±SD. **, *P* < 0.01; ***, *P* < 0.001, ns, no significant difference, One-way ANOVA.


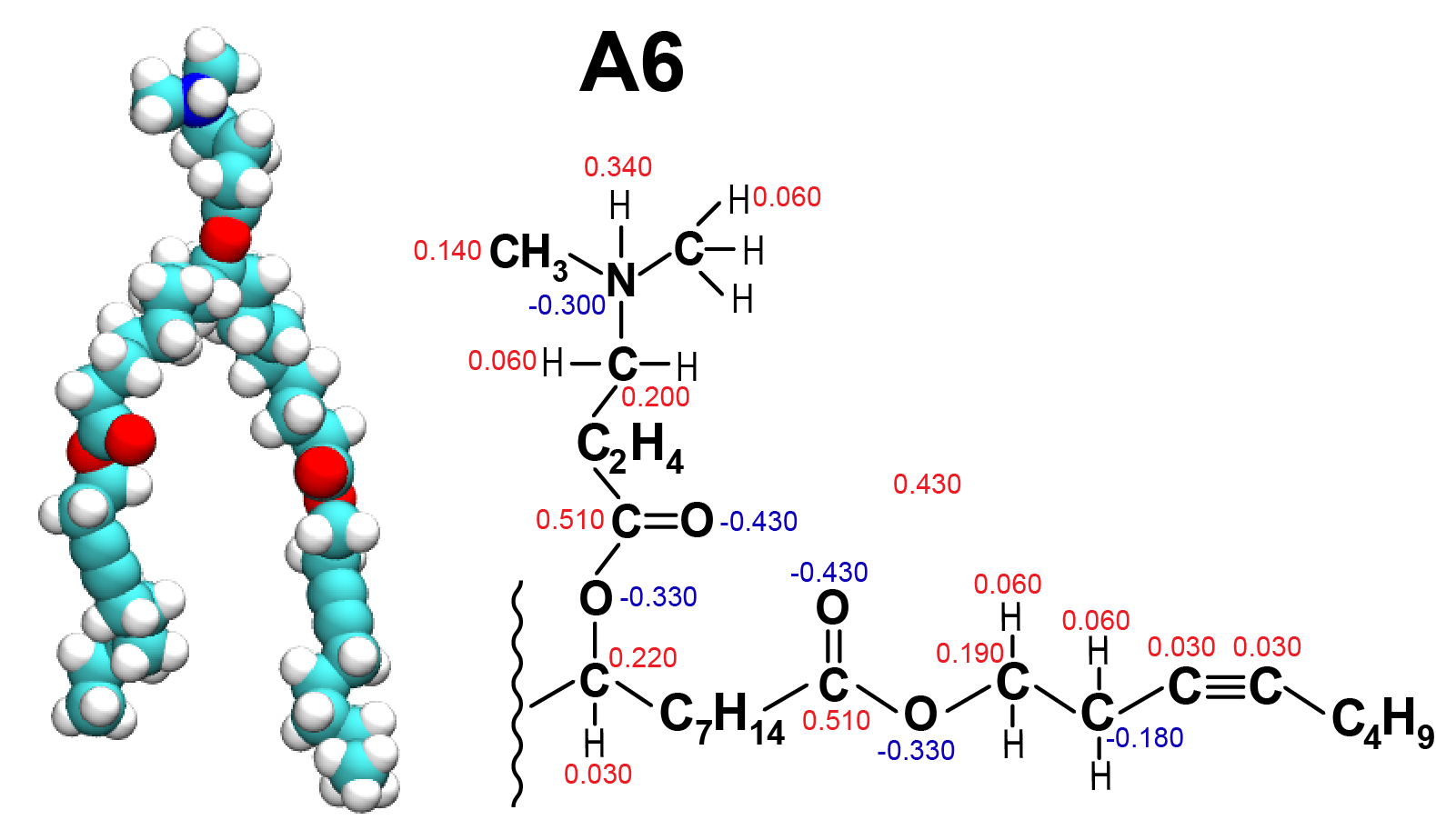


**Supplementary Figure 9. Topology and partial charge of ionized A6 lipid for molecular dynamic (MD) simulation.** Penal on the left: schematic of lipid A6 drawn as Van der Waals (VDW) spheres. Penal on the right: partial charge of functional groups in A6 in OPLS all-atomistic force field. Since A6 has two same hydrophobic alkyne tails, only one of them is shown. Omitted hydrogen atoms have partial charges of 0.06 *e* whereas omitted carbon atoms have partial charges of either -0.12 *e* or -0.18 *e* depends on the number of hydrogen atoms bonded to them.


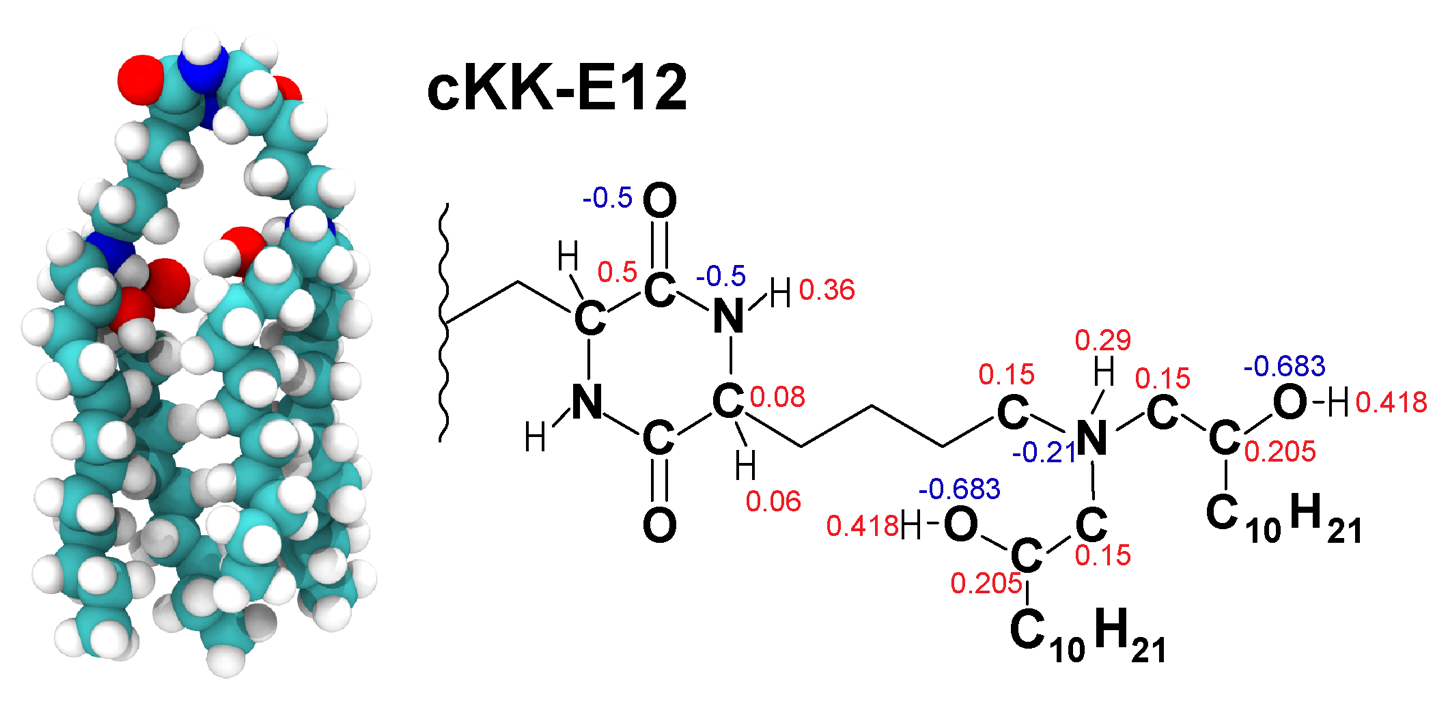


**Supplementary Figure 10. Topology and partial charge of ionized cKK-E12 lipid for MD simulation.** Penal on the left: schematic of cKK-E12 represented by VDW spheres. Penal on the right: partial charge of functional groups in cKK-E12 in OPLS all-atomistic force field. Since the molecular structure of cKK-E12 is symmetric, only right half is shown. Omitted hydrogen atoms have partial charges of 0.06 *e* whereas omitted carbon atoms have partial of either -0.12 *e* or -0.18 *e* depends on the number of hydrogen atoms bonded to them.


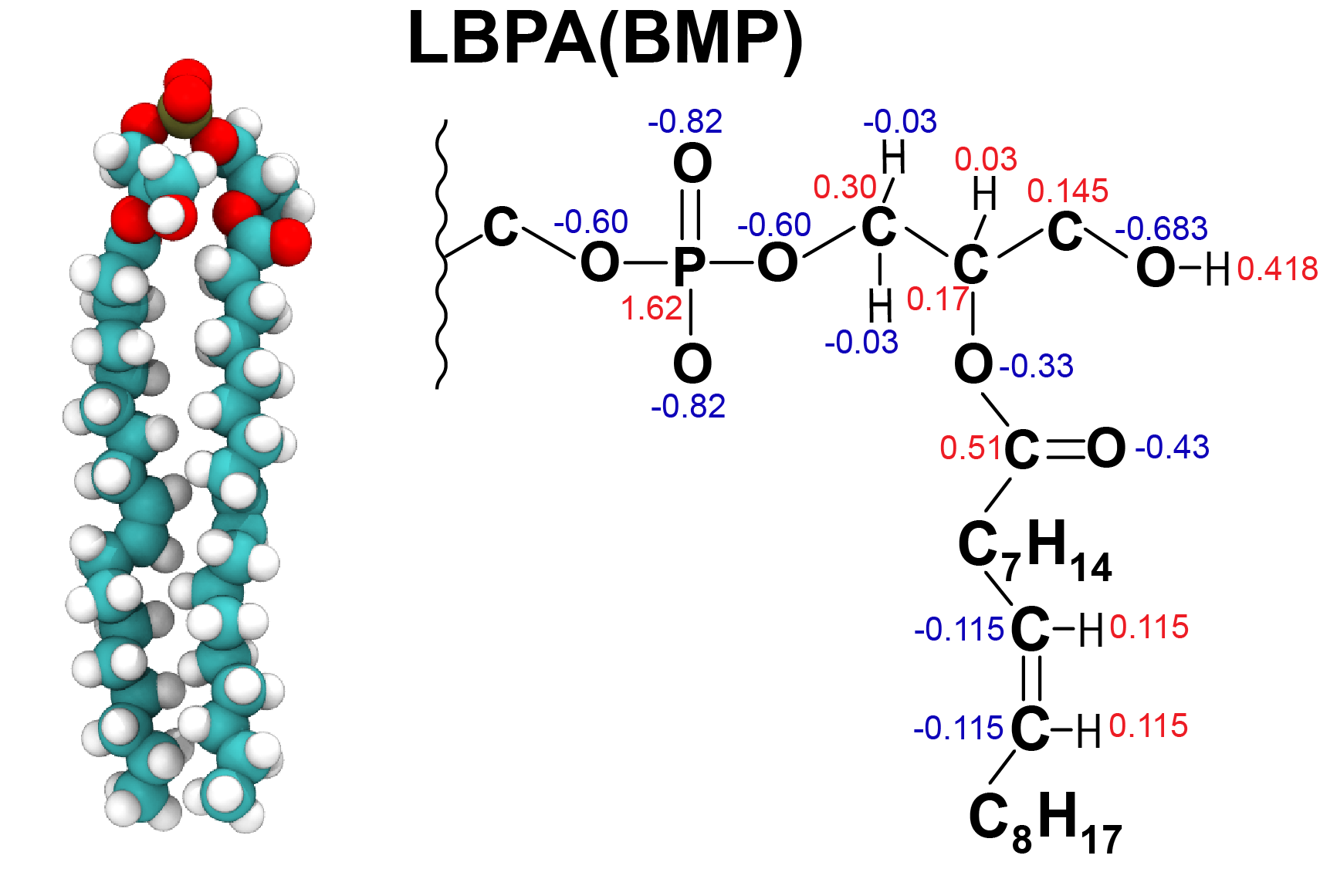


**Supplementary Figure 11. Topology and partial charge of LBPA (BMP) for MD simulation.** Penal on the left: schematic of LBPA represented by VDW spheres. Penal on the right: partial charge of functional groups in LBPA in OPLS all-atomistic force field. Right half of LBPA is shown. Omitted hydrogen atoms have partial charges of 0.06 *e* whereas omitted carbon atoms have partial charges of either -0.12 *e* or -0.18 *e* depends on the number of hydrogen atoms bonded to them.

**
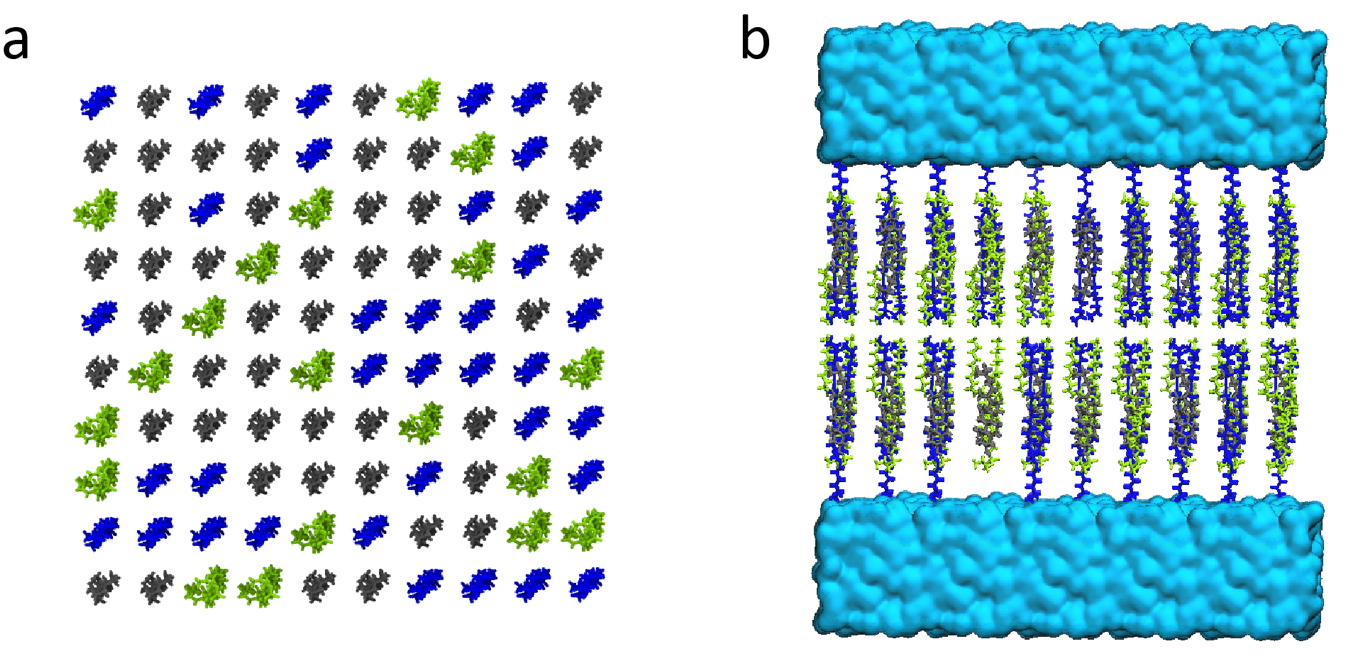
**

**Supplementary Figure 12. Building of A6 and cKK-E12 bilayer membrane.** (a) A6, DOPE, and cholesterol are randomly aligned in a 10 by 10 grid (top view). (b) Water molecules are added to the membrane (lateral view) before NPT simulation.

**
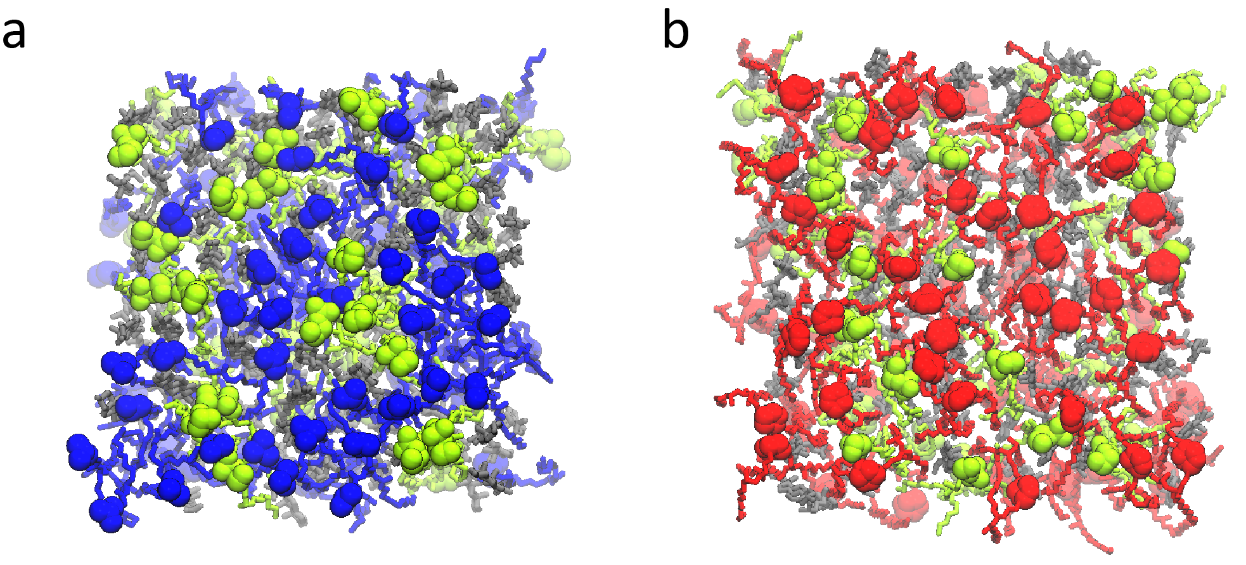
**

**Supplementary Figure 13. Snapshots (top view) of the relaxed membranes after 100 ns simulation.** (a) A6 membrane. (b) cKK-E12 membrane. Color code: A6 is presented in blue, cKK-E12 in red, DOPE and DOPC in green, and cholesterol in gray.

**
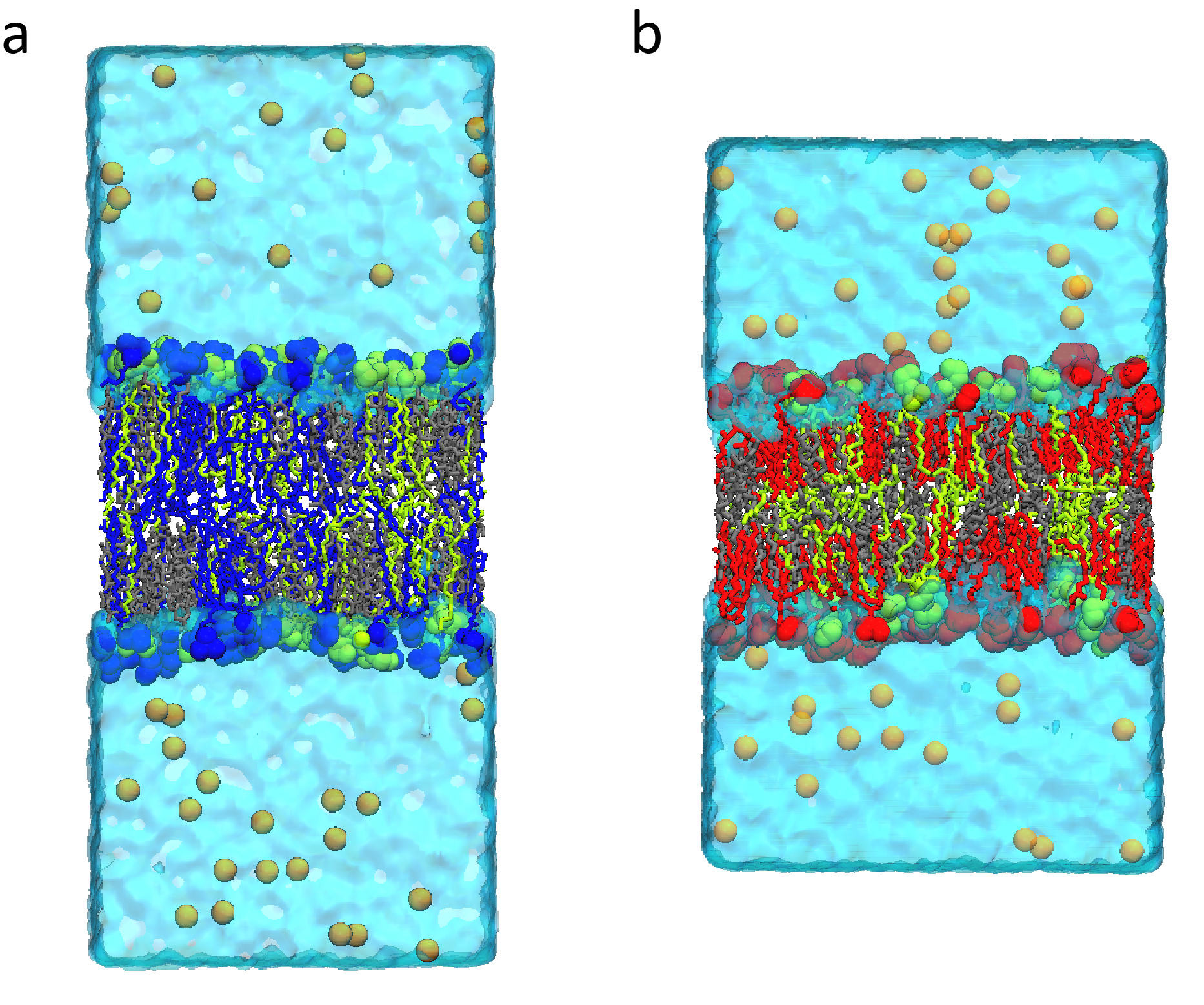
**

**Supplementary Figure 14. Snapshots (lateral view) of relaxed membranes after 100 ns simulation.** (a) A6 membrane. (b) cKK-E12 membrane. Color code: A6 is presented in blue, cKK-E12 in red, DOPE and DOPC in green, and cholesterol in gray.

**
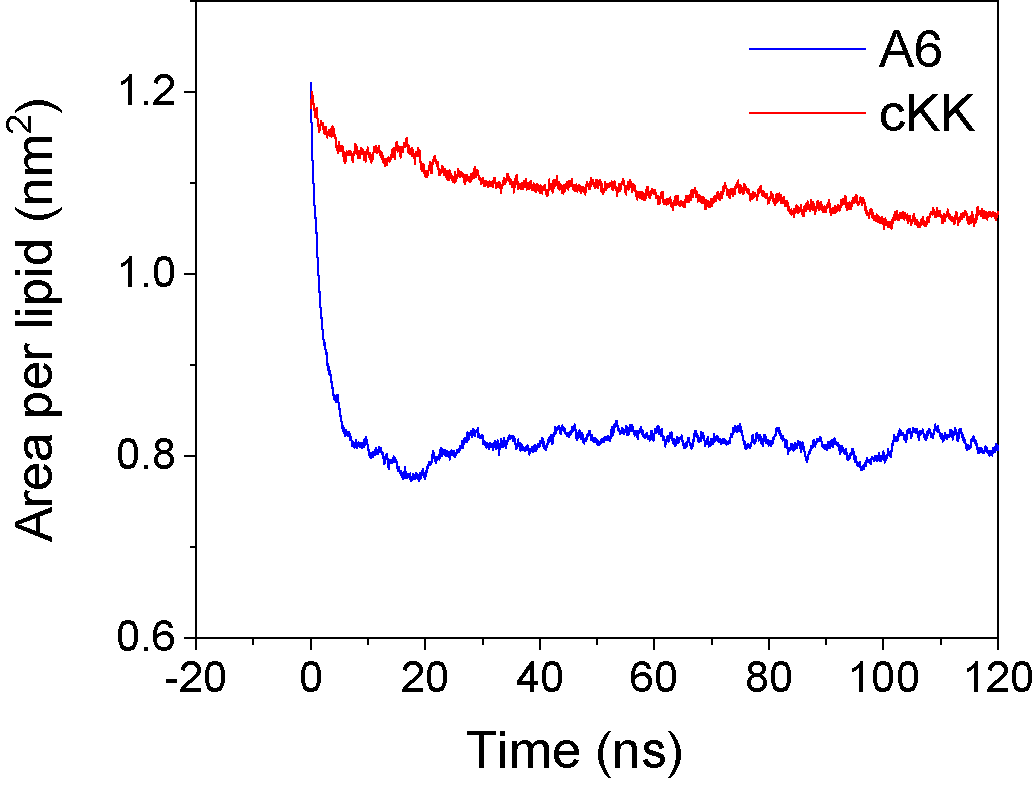
**

**Supplementary Figure 15. Area per lipid of A6 and cKK-E12 membranes for 120 ns simulations.**


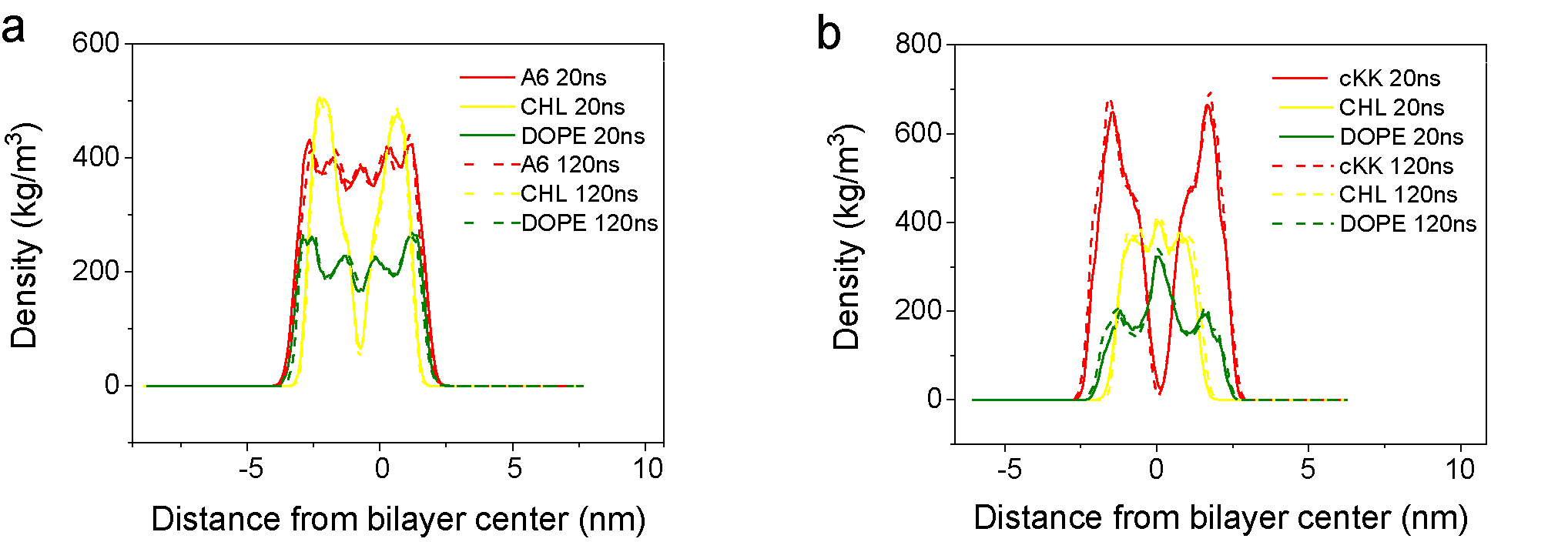


**Supplementary Figure 16. Lipid density profiles of A6 membrane (a) and cKK-E12 membrane (b) after 20 ns equilibration and 100 ns production run.**


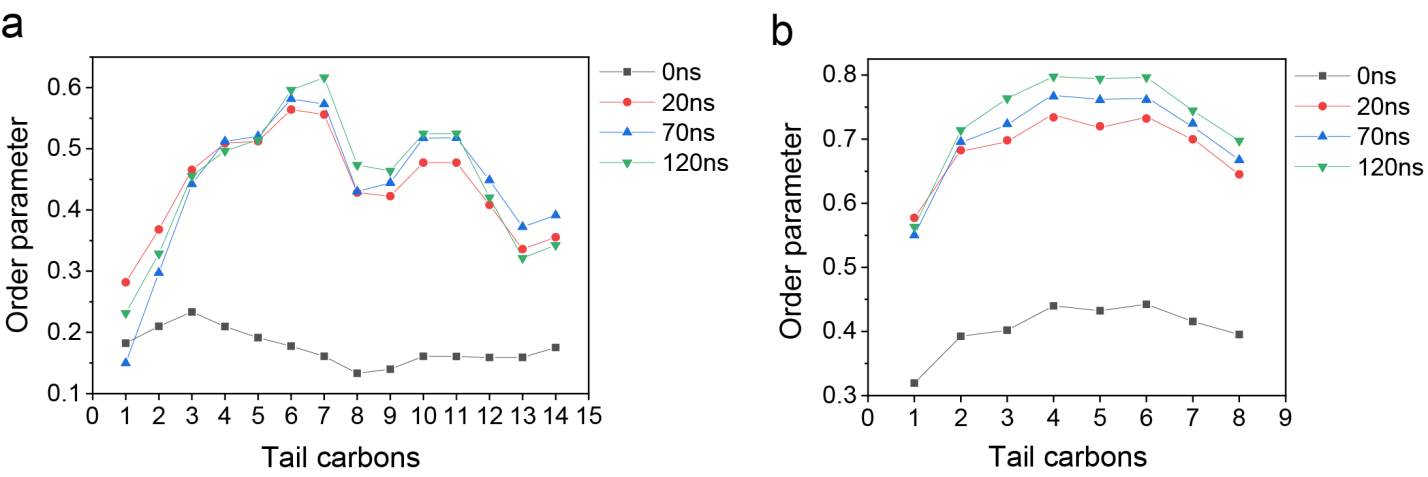


**Supplementary Figure 17. Lipid tails second order parameter of A6 membrane (a) and cKK-E12 membrane (b) at 20 ns, 70 ns, and 120 ns simulation time.**


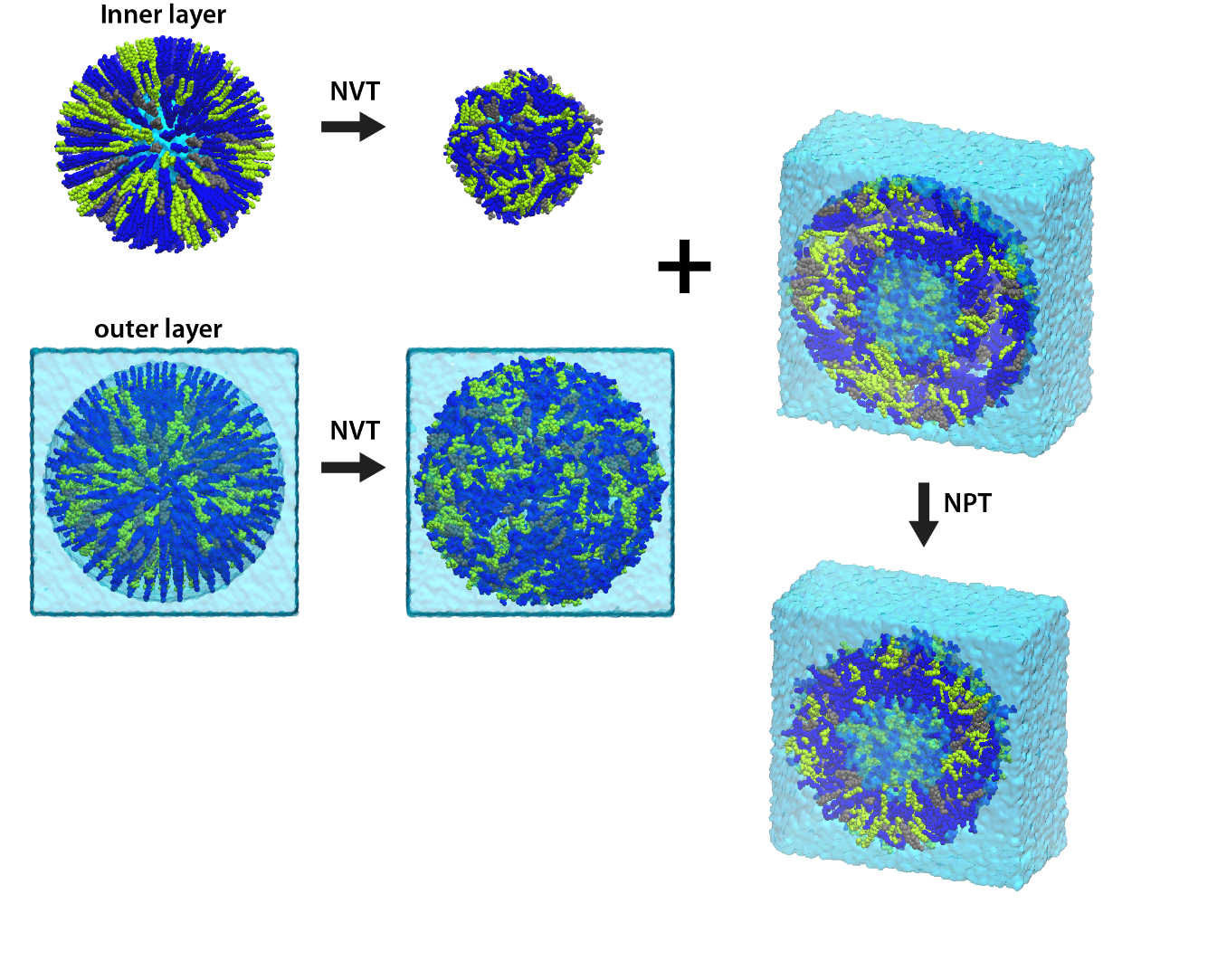


**Supplementary Figure 18. Building of vesicles (A6, cKK-E12 and endosomal vesicles) for MD simulation.** For the inner layer of the vesicle, A6 or cKK-E12 or LBPA, DOPE and cholesterol with a predefined ratio (for building of A6, cKK-E12 and endosomal vesicle separately) were aligned inwardly on a spherical surface with a water core (5 nm diameter). The system was equilibrated using canonical NVT ensemble to keep the dimension of the box fixed. For the outer layer, the lipid components with calculated amount were aligned outwardly on a spherical surface with a water shell (16×16×16 nm^3^) surrounding the lipids. The system was equilibrated in a NVT ensemble. Then the outer layer and the inner layer system were combined (with a vacuum gap between the two layers) and equilibrated in a isothermal-isobaric NPT ensemble. As the system was condensed and the vacuum gap was eliminated, a complete vesicle was formed with a diameter of 14.3 nm.

**
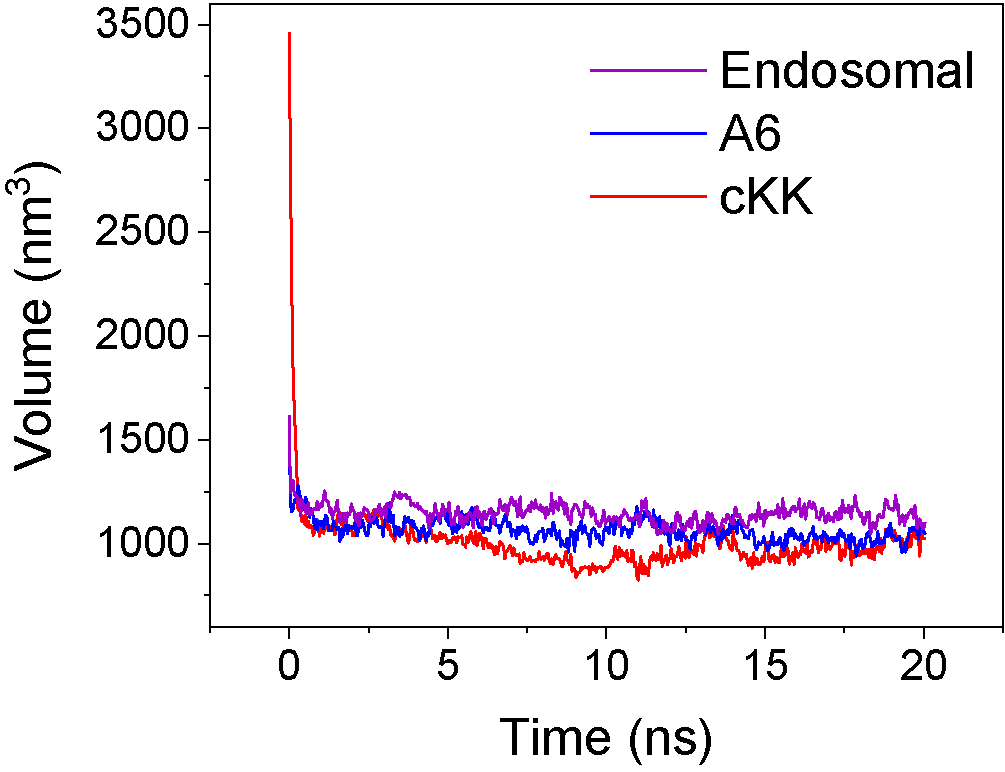
**

**Supplementary Figure 19. Estimated volume of A6, cKK-E12, and endosomal vesicle during NPT equilibration after combing inner and outer vesicles.**


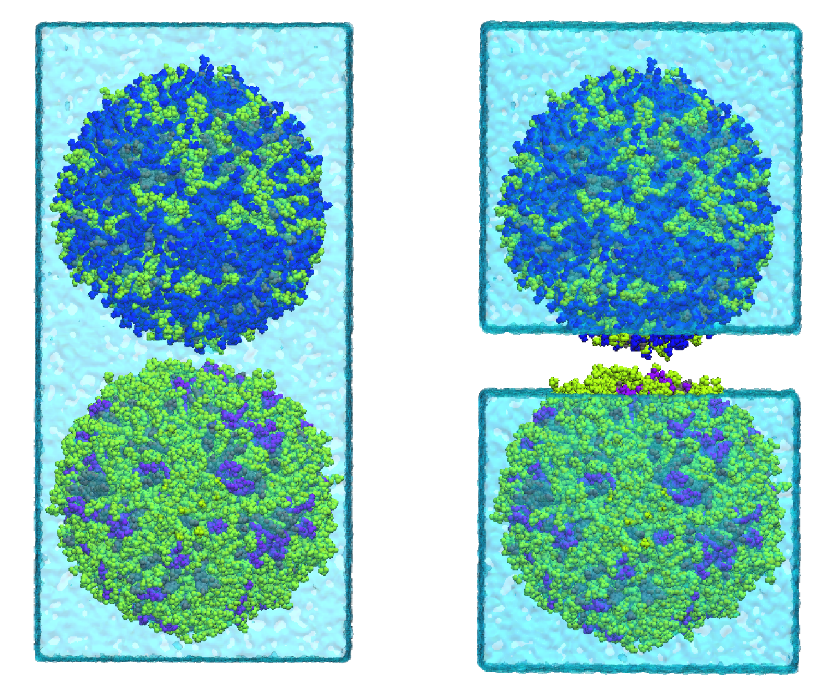


**Supplementary Figure 20. Initiation of fusion simulation.** Different lipid vesicles (A6 and endosomal vesicles; cKK-E12 and endosomal vesicles) were randomly embedded in water. Water molecules (3 nm thick) in the middle are removed to facilitate initial contact between A6 or cKK-E12 vesicle (top) with endosomal vesicle (bottom). System has a dimension of 16×16×32 nm^3^ and contains approximately 160,000 water molecules.


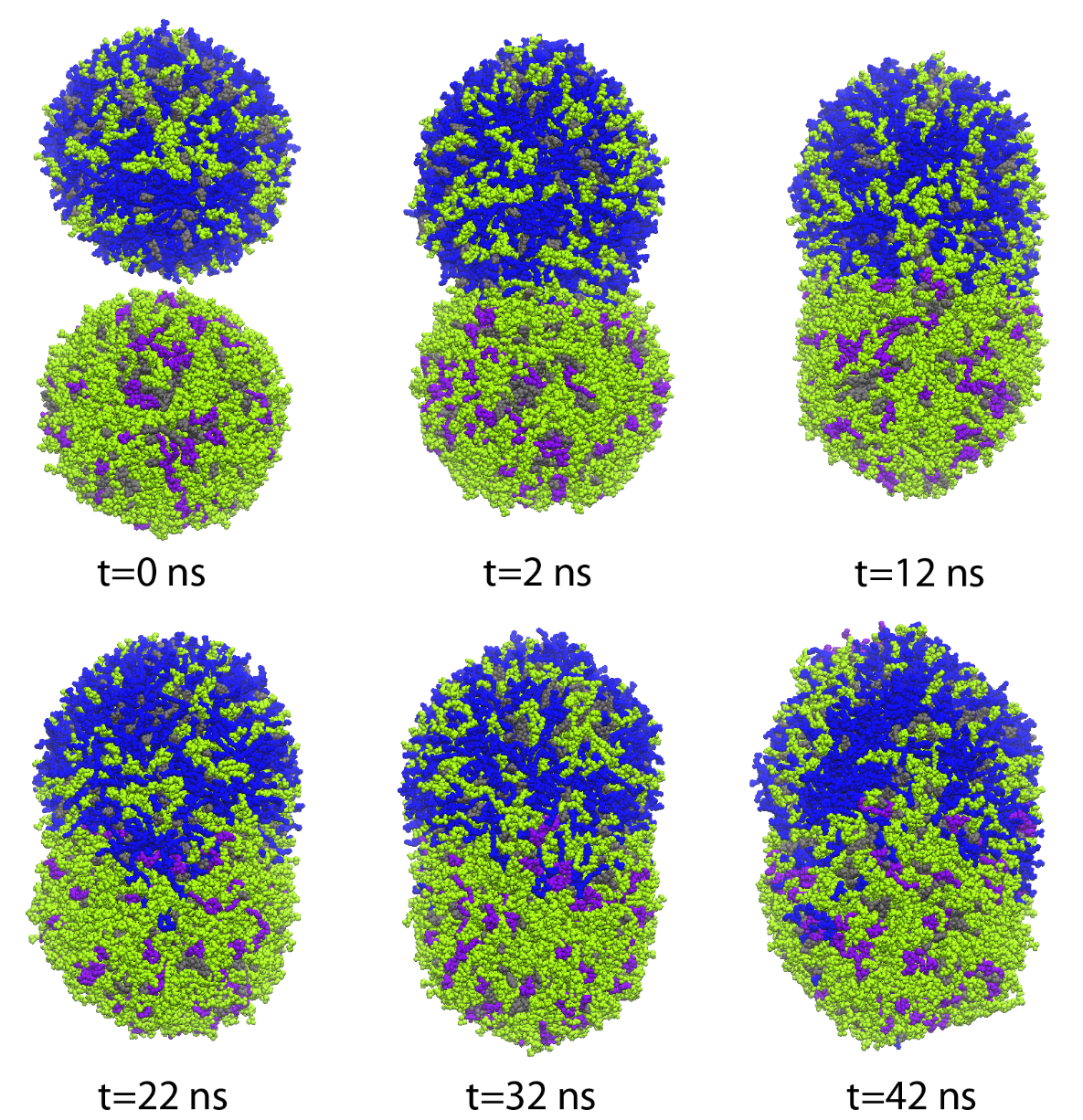


**Supplementary Figure 21. Snapshots of fusion between A6 vesicle and endosomal vesicle by all-atom MD simulations (OPLS with TIP4P water model).** A6 vesicle is located on the top, endosomal vesicles is located at the bottom. Water at the neck region was removed at the beginning of the simulation (n = 0s). A6 lipid is presented in blue, DOPE and DOPC in green, LBPA in purple, and cholesterol in gray. Water is omitted for clarity.


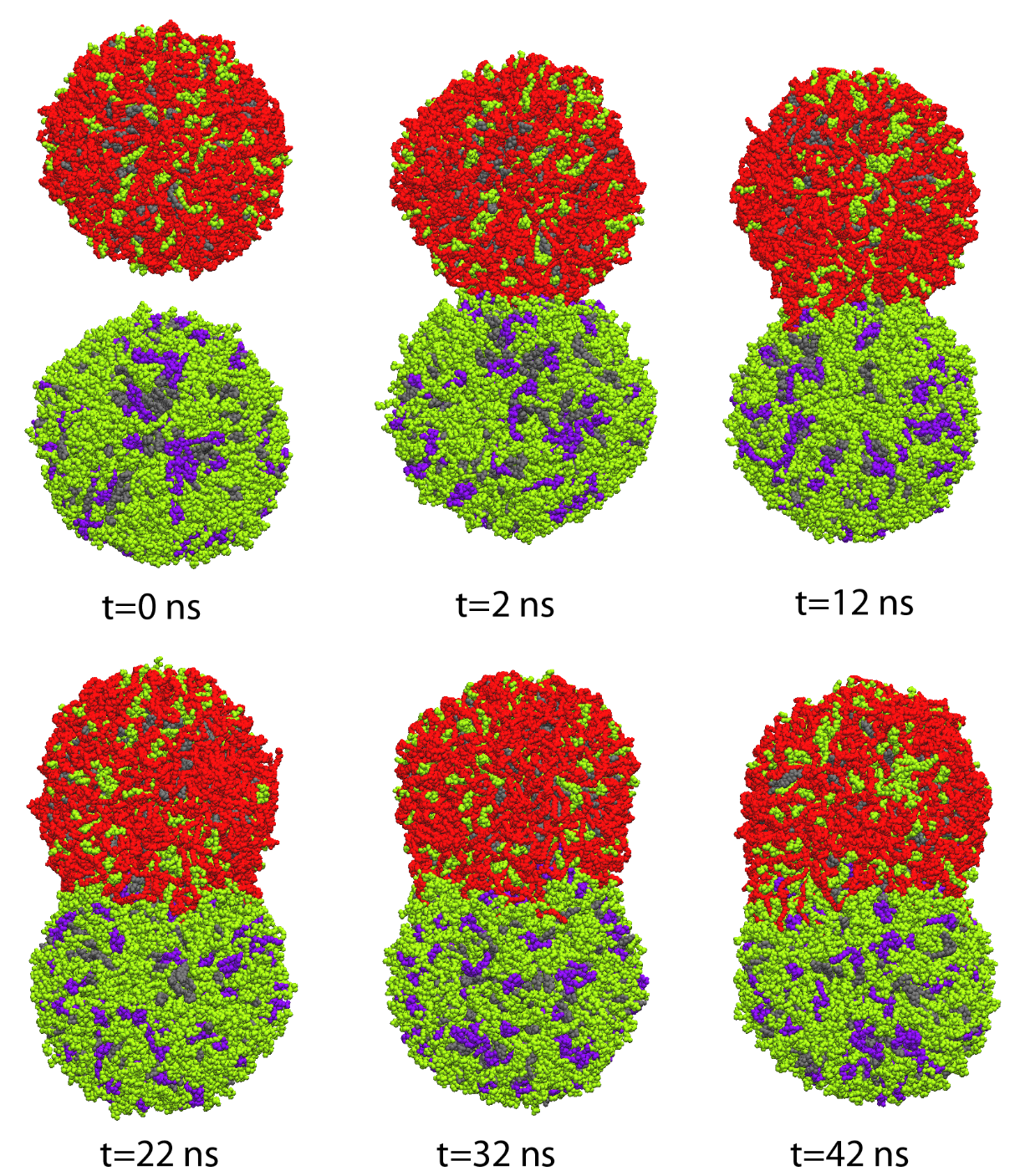


**Supplementary Figure 22. Snapshots of fusion between cKK-E12 vesicle and endosomal vesicle by all-atom MD simulations (OPLS with TIP4P water model).** cKK-E12 vesicle is located on the top, endosomal vesicles is located at the bottom. Water at the neck region was removed at the beginning of the simulation (n = 0s). cKK-E12 lipid is presented in red, DOPE and DOPC in green, LBPA in purple, and cholesterol in gray. Water is omitted for clarity.


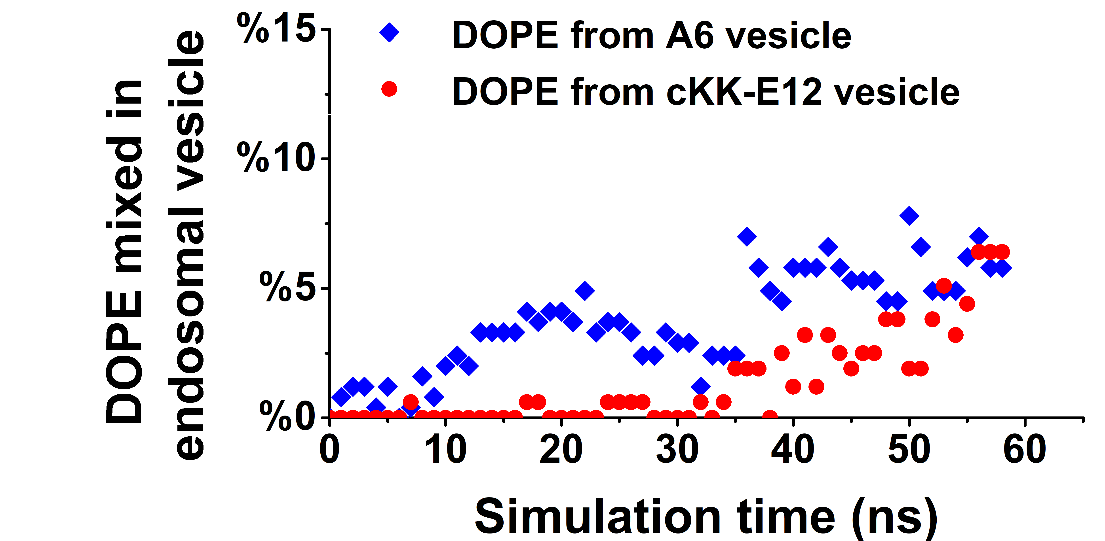


**Supplementary Figure 23. Percentile of DOPE (from A6 or cKK-E12 vesicles) mixed in endosomal vesicles during fusion simulation.** The boundary separates the merging vesicle that located at the center of two vesicles.

**
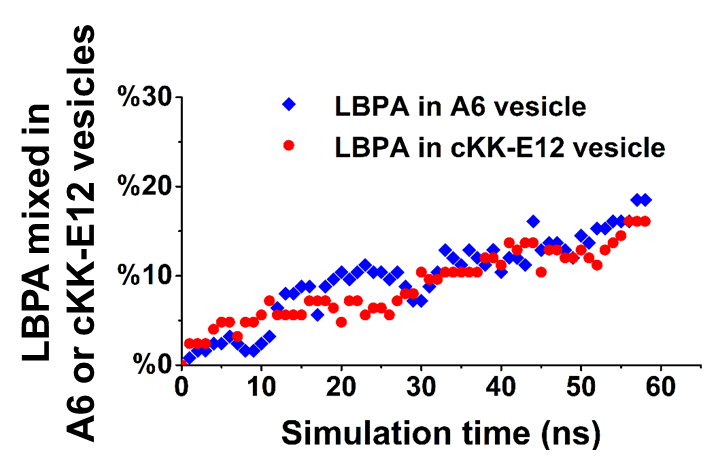
**

**Supplementary Figure 24. Percentile of LBPA mixed in A6 or cKK-E12 vesicles during fusion simulation.** The boundary separates the merging vesicle is located at the center of mass of the two vesicles.


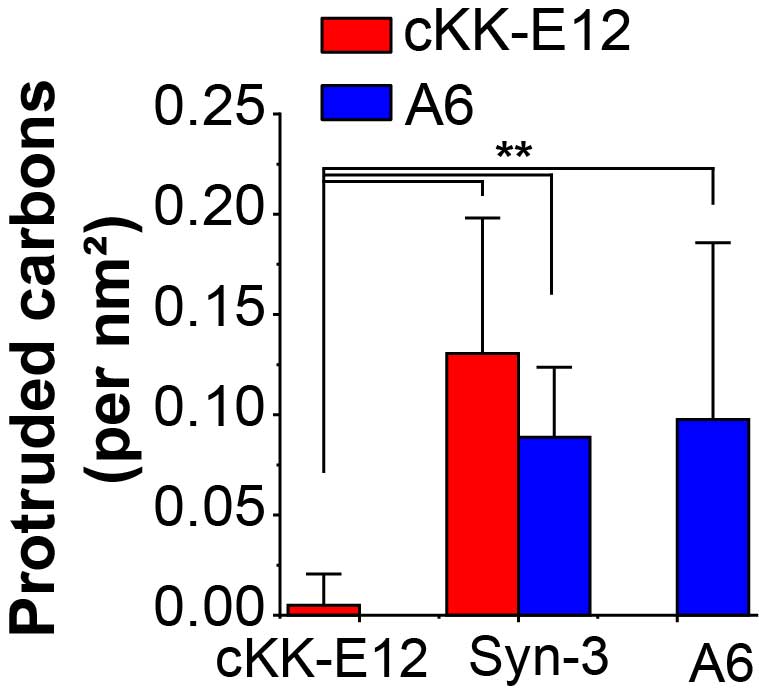


**Supplementary Figure 25. MD calculations of lipid tail protrusion of cKK-E12 and A6 in singular or synergistic membranes.** Data are collected from the last 20 ns of the trajectory files with an interval of 0.02 ns (n=1000). Data are presented as mean±SD. ** P < 0.01, Student’s T-test.

**
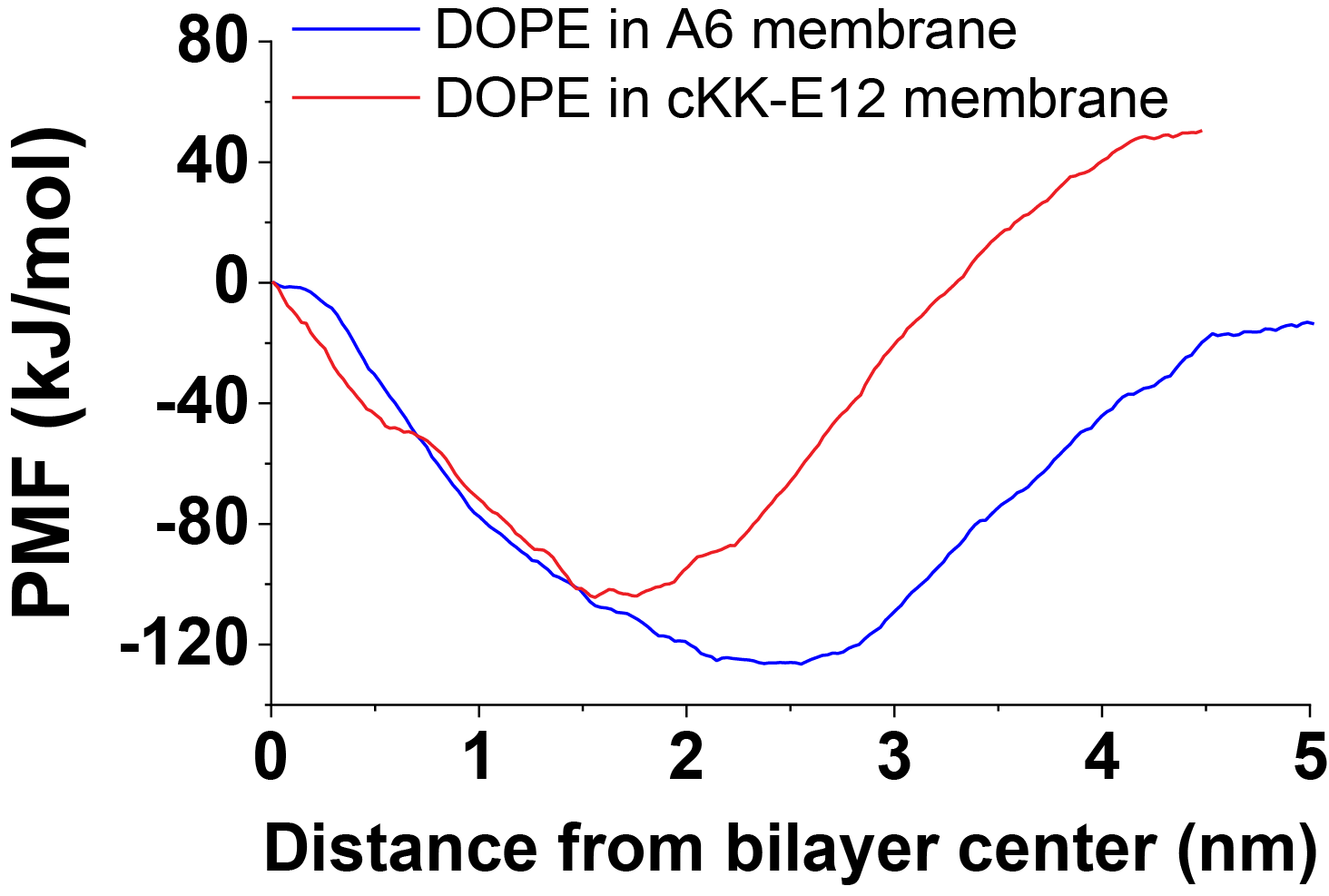
**

**Supplementary Figure 26. Free energy profile of DOPE sprouting and flip-flop action in A6 and cKK-E12 membranes.** Shaded area are 5% error of data.

**
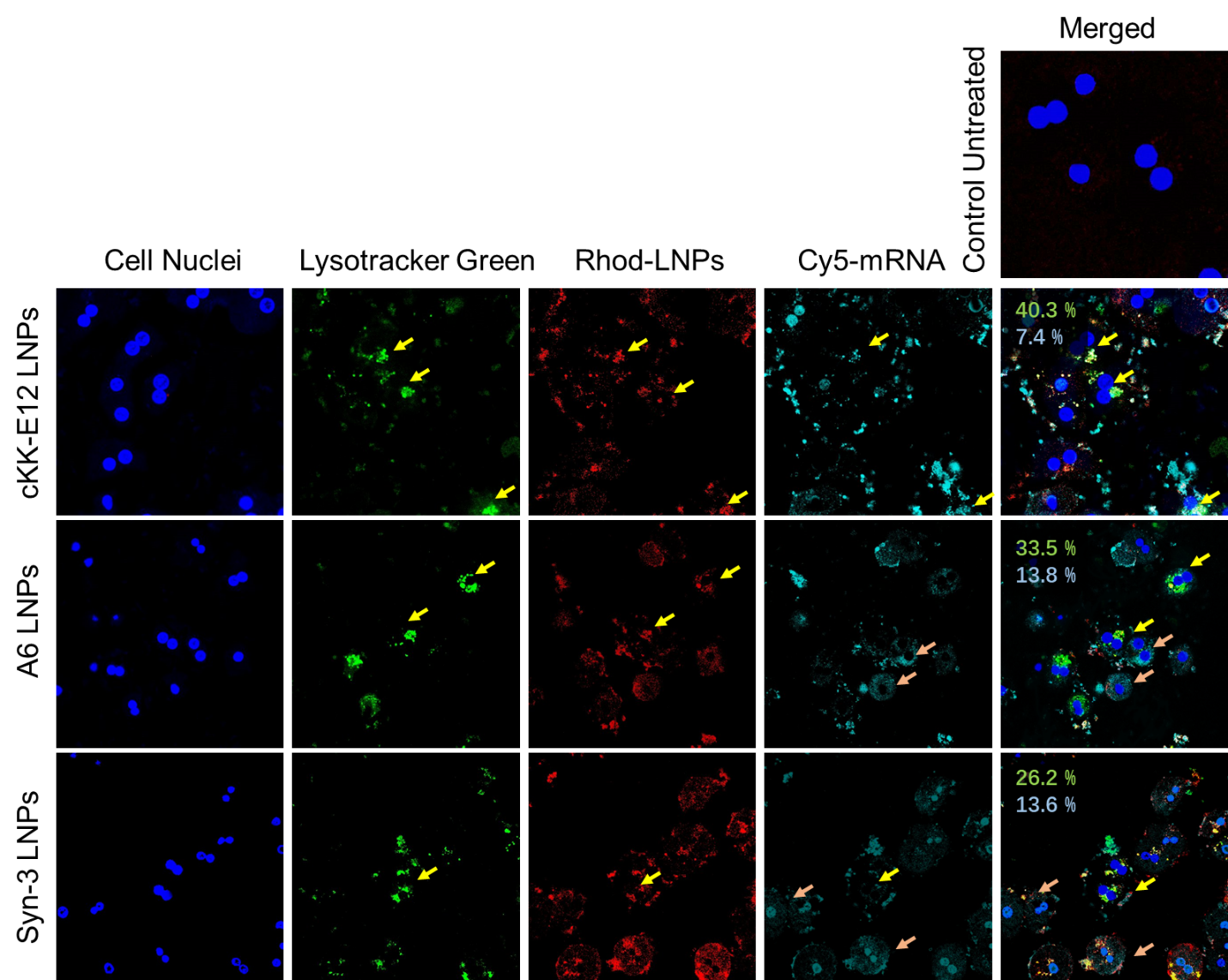
**

**Supplementary Figure 27. A control of untreated primary hepatocytes and co-staining of lysotracker with fluorescence labeled LNPs.** LNPs were encapsulated with Cy5-mRNA, labeled with Rhod-PE lipid, and incubate with primary hepatocytes for 1h. The cells were then stained with lysotracker, washed with PBS three times, and imaged by confocal microscope. Yellow arrows indicated trapping of LNPs in endo-lysosomes. Orange arrows pointed out the release of mRNA into cytoplasm. Numbers in green is the % of Cy5-mRNA co-localized with lysotracker-green (trapped in the endo-lysosomes). Numbers in cyan is the % of Cy5-mRNA distributed (disseminated) in the cytoplasm.

**
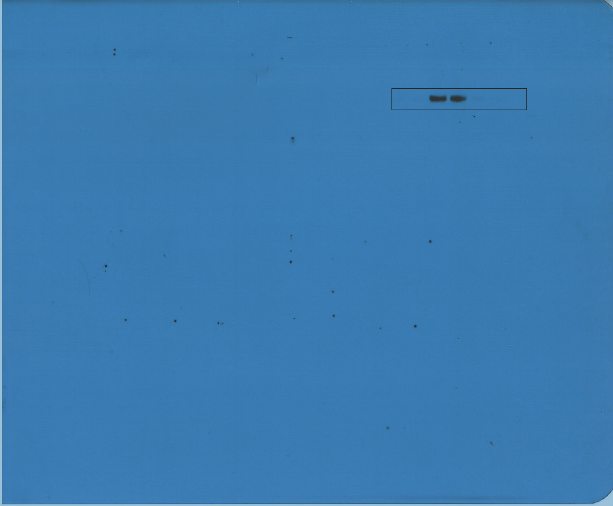
**

**Supplementary Figure 28. A raw western blot image of Lamp-1 (uncropped blot for Fig. 3n)**

*
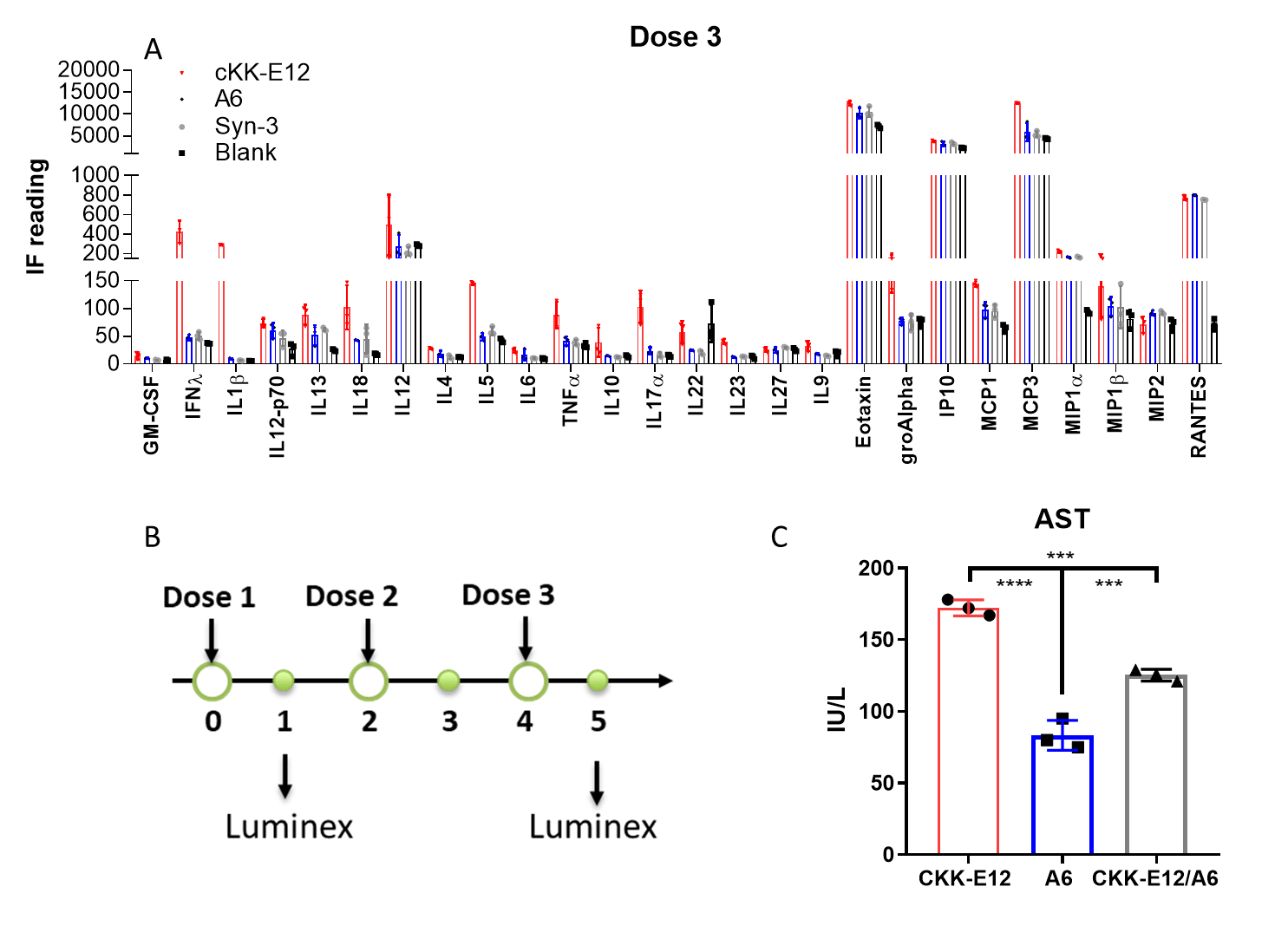
*

**Supplementary Figure 29. Toxicity evaluation after multiple dosing of LNPs.** A. the multi-plex cytokine assay of blank LNPs after 3 injections of LNPs (n = 4, equivalent to 1.5 mg/kg mRNA LNPs). B. An illustration of the dosing regimen. C. AST measurement after 3 doses of mRNA containing LNPs (n = 4, 1.5 mg/kg mRNA). Data are presented as mean ± SD. *** *P* <0.001, **** *P* < 0.0001, One-way ANOVA.


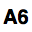


**Supplementary Figure 30. Synthesis route of A6**

**Reference**

1. Knecht, V. & Marrink, S.-J. Molecular dynamics simulations of lipid vesicle fusion in atomic detail. *Biophys. J.* **92**, 4254–4261 (2007).
