# Supplementary material for: Synergistic lipid compositions for albumin receptor mediated delivery of mRNA to the liver
Source: Nat Commun. 2020 May 15;11:2424. doi: 10.1038/s41467-020-16248-y (PMC7229004; doi:10.1038/s41467-020-16248-y)
Supplement: Supplementary file 2 — Reporting Summary [file 41467_2020_16248_MOESM2_ESM.pdf]

nature research

Corresponding author(s): Daniel G Anderson

Last updated by author(s): Mar 1, 2020

Reporting Summary

Nature Research wishes to improve the reproducibility of the work that we publish. This form provides structure for consistency and transparency in reporting. For further information on Nature Research policies, see Authors & Referees and the Editorial Policy Checklist .

Statistics

For all statistical analyses, confirm that the following items are present in the figure legend, table legend, main text, or Methods section.

n/a	Confirmed	
☐	☒	The exact sample size (*n*) for each experimental group/condition, given as a discrete number and unit of measurement
☐	☒	A statement on whether measurements were taken from distinct samples or whether the same sample was measured repeatedly
☐	☒	The statistical test(s) used AND whether they are one- or two-sided<br>*Only common tests should be described solely by name; describe more complex techniques in the Methods section.*
☒	☐	A description of all covariates tested
☒	☐	A description of any assumptions or corrections, such as tests of normality and adjustment for multiple comparisons
☐	☒	A full description of the statistical parameters including central tendency (e.g. means) or other basic estimates (e.g. regression coefficient) AND variation (e.g. standard deviation) or associated estimates of uncertainty (e.g. confidence intervals)
☒	☐	For null hypothesis testing, the test statistic (e.g. *F*, *t*, *r*) with confidence intervals, effect sizes, degrees of freedom and *P* value noted<br>*Give P values as exact values whenever suitable.*
☒	☐	For Bayesian analysis, information on the choice of priors and Markov chain Monte Carlo settings
☒	☐	For hierarchical and complex designs, identification of the appropriate level for tests and full reporting of outcomes
☒	☐	Estimates of effect sizes (e.g. Cohen's *d*, Pearson's *r*), indicating how they were calculated

Our web collection on statistics for biologists contains articles on many of the points above.

Software and code

Policy information about availability of computer code

Data collection	No computer code was used
Data analysis	Statistical analysis was performed using excel and GraphPad Prism

For manuscripts utilizing custom algorithms or software that are central to the research but not yet described in published literature, software must be made available to editors/reviewers. We strongly encourage code deposition in a community repository (e.g. GitHub). See the Nature Research guidelines for submitting code & software for further information.

Data

Policy information about availability of data

All manuscripts must include a data availability statement. This statement should provide the following information, where applicable:

- Accession codes, unique identifiers, or web links for publicly available datasets
- A list of figures that have associated raw data
- A description of any restrictions on data availability

Authors can confirm that all relevant data are included in the paper and/or its supplementary information files

Field-specific reporting

Please select the one below that is the best fit for your research. If you are not sure, read the appropriate sections before making your selection.

☒ Life sciences ☐ Behavioural & social sciences ☐ Ecological, evolutionary & environmental sciences

For a reference copy of the document with all sections, see nature.com/documents/nr-reporting-summary-flat.pdf

October 2018

Life sciences study design

All studies must disclose on these points even when the disclosure is negative.

Sample size	Sample sizes were determined to allow the statistical significance of differences of 50% or greater, and according to similar studies conducted in the field. The specific sample size required is depended on the experiments
Data exclusions	No data were excluded
Replication	All in vitro experiments were conducted at least three or four times. All in vivo experiments were conducted at least five times or more.
Randomization	Mice were divided into different groups randomly
Blinding	Blinded study was used for some of the animal studies when the formulations appear similar (but not blinded for control group)

Reporting for specific materials, systems and methods

We require information from authors about some types of materials, experimental systems and methods used in many studies. Here, indicate whether each material, system or method listed is relevant to your study. If you are not sure if a list item applies to your research, read the appropriate section before selecting a response.

Materials & experimental systems

n/a	Involved in the study	
☐	☒	Antibodies
☐	☒	Eukaryotic cell lines
☒	☐	Palaeontology
☐	☒	Animals and other organisms
☒	☐	Human research participants
☒	☐	Clinical data

Methods

n/a	Involved in the study	
☒	☐	ChIP-seq
☐	☒	Flow cytometry
☒	☐	MRI-based neuroimaging

Antibodies

Antibodies used	Rabbit mAb LAMP1 (D2D11)XP Rabbit mAB #9091 (cell signaling); Goat Anti-Rabbit IgG H&L (HRP, cell signaling)
Validation	Cell signaling has validated the antibodies (Data were shown on their website)

Eukaryotic cell lines

Policy information about cell lines

Cell line source(s)	Primary mouse hepatocytes
Authentication	cell line was confirmed by flow cytometry
Mycoplasma contamination	No mycoplasma contamination
Commonly misidentified lines (See ICLAC register)	No

Animals and other organisms

Policy information about studies involving animals; ARRIVE guidelines recommended for reporting animal research

Laboratory animals	Six-week-old male C57BL/6 mice from Charles River were used for the adenine-induced renal anemia model and the separation of primary hepatocytes
Wild animals	No
Field-collected samples	No
Ethics oversight	All experimental procedures were ethically approved and performed under the guidelines of the Division of Comparative Medicine by Massachusetts Institute of Technology

Note that full information on the approval of the study protocol must also be provided in the manuscript.

Flow Cytometry

Plots

Confirm that:

- [x] The axis labels state the marker and fluorochrome used (e.g. CD4-FITC).
- [x] The axis scales are clearly visible. Include numbers along axes only for bottom left plot of group (a 'group' is an analysis of identical markers).
- [ ] All plots are contour plots with outliers or pseudocolor plots.
- [x] A numerical value for number of cells or percentage (with statistics) is provided.

Methodology

Sample preparation	Primary hepatocytes were isolated from B6 mice according to the method described in the method part
Instrument	BD LSRFFortessa
Software	FlowJo
Cell population abundance	For In vitro study, ~10,000 relevant hepatocytes were acquired. For in vivo study, ~5000 cells were collected for the lowest components (collect as much as possible)
Gating strategy	cells were gated based on FSC/SSC, single cells FSC-H and FSC-A. Dead cells were excluded.

- [x] Tick this box to confirm that a figure exemplifying the gating strategy is provided in the Supplementary Information.
